# Rethinking Salt in Cheese: Advances, Challenges, and Strategies for Sodium Reduction

**DOI:** 10.3390/foods15193395

**Published:** 2026-09-23

**Authors:** Charikleia Tsanasidou, Loulouda Bosnea, Antonia Terpou

**Affiliations:** 1 Department of Agricultural Development, Agri-Food and Natural Resources Management, School of Agricultural Development, Nutrition & Sustainability, National and Kapodistrian University of Athens, Evripos Campus, 34400 Athens, Greece; xaroulatsan@gmail.com; 2 Dairy Research Department, Hellenic Agricultural Organization-DIMITRA, Katsikas, 45221 Ioannina, Greece; louloudabosnea@gmail.com or bosnea@elgo.gr

**Keywords:** reduced-sodium cheese, salt substitutes, potassium chloride, microbial safety, cheese ripening, sensory quality

## Abstract

Sodium chloride (NaCl) is a multifunctional component of cheese manufacture that contributes to flavor, microbial control, moisture regulation, texture formation, product safety, and shelf stability. However, the association between excessive dietary sodium intake, elevated blood pressure, and cardiovascular disease has increased demand for reduced-sodium foods. Cheese reformulation is particularly challenging because reducing NaCl may simultaneously modify water activity, microbial ecology, acidification, proteolysis, lipolysis, texture, flavor development, and sensory acceptance. Accordingly, the purpose of this review is to critically evaluate the scientific evidence concerning direct NaCl reduction, partial replacement with mineral salts, flavor-enhancement approaches, modified salting and processing technologies, and complementary preservation strategies in cheese. Particular attention is given to the mechanisms through which these interventions affect microbial succession, ripening biochemistry, rheological properties, safety, shelf life, and consumer acceptance. The review also evaluates the limitations affecting industrial implementation, including process reproducibility, regulatory compliance, mineral accumulation in recirculated brines, ingredient costs, and environmental considerations. Current evidence indicates that no single strategy is universally applicable. Instead, safe and acceptable sodium reduction requires cheese-specific combinations of salt replacement, process control, microbial management, and sensory optimization.

## 1. Introduction

Salt is often regarded primarily as a flavoring and preservation ingredient; in cheese, however, it functions as a critical formulation and processing variable. Sodium chloride (NaCl) influences not only saltiness but also water activity (α_w_), microbial growth, enzyme activity, moisture distribution, protein hydration, texture development, and shelf stability [1,2]. Chemically, NaCl contains approximately 39.3% (*w*/*w*) sodium and 60.7% (*w*/*w*) chloride; therefore, 2.54 g of NaCl provides approximately 1 g of sodium [3]. Although the terms “salt reduction” and “sodium reduction” are frequently used interchangeably, they are not equivalent. Sodium content can be reduced through partial replacement of NaCl with non-sodium salts without necessarily decreasing the total concentration of added mineral salts. This distinction is particularly important when evaluating reformulation strategies and interpreting their technological effects [4,5].

High sodium intake remains an important global public-health concern because of its established association with elevated blood pressure and increased cardiovascular disease risk. The World Health Organization (WHO) recommends limiting sodium intake to less than 2 g/day, corresponding to approximately 5 g of salt. Nevertheless, mean global sodium intake has been estimated at approximately 4.3 g/day, more than twice the recommended level, and the WHO has established a target of reducing population sodium intake by 30% by 2030 [6]. Because a substantial proportion of dietary sodium is supplied by manufactured foods rather than salt added during domestic cooking, reformulation of commonly consumed food categories is considered an essential component of population-wide sodium reduction [7]. Within the European Union, nutrition claims concerning sodium and salt are governed by Regulation (EC) No 1924/2006. The claim “reduced sodium” or “reduced salt” may be used when the sodium content, or the equivalent value for salt, is at least 25% lower than that of comparable foods in the same category. This is an exception to the general 30% reduction required for most other nutrients. A statement such as “50% less sodium” is not a separate regulatory category but may quantify the achieved reduction when the conditions for a comparative nutrition claim are satisfied. The comparison must relate to the same quantity of food and consider a range of foods within the same category; it is not based on the population reference intake or on an assumed natural sodium content. Regulation (EC) No 1924/2006 additionally defines “low sodium/salt” as no more than 0.12 g sodium, or the equivalent value for salt, per 100 g or 100 mL of food [8].

Cheese is a nutrient-dense fermented food that provides high-quality proteins, lipids, calcium, phosphorus, and fat-soluble vitamins. At the same time, several cheese categories can contribute appreciably to dietary sodium intake because NaCl is added during dry salting, brining, curd salting, or processing. Sodium concentration varies considerably among cheese varieties according to moisture content, manufacturing procedure, curd dimensions, salting method, brine concentration, ripening conditions, and intended shelf life [5]. Brined and processed cheeses generally contain higher sodium concentrations than many fresh cheeses, although substantial variation may also occur among products belonging to the same cheese category [9]. Consequently, sodium reduction cannot be addressed through a single formulation target applicable to all cheeses.

The technological importance of NaCl extends beyond its concentration in the final product. Salt-in-moisture (S/M) content, spatial salt distribution, and the rate of salt diffusion through the cheese matrix determine the actual exposure of microorganisms and enzymes to NaCl during ripening [10,11]. Immediately after brining or surface salting, pronounced concentration gradients may develop between the surface and center of the cheese. These gradients subsequently influence microbial succession, acidification, proteolysis, lipolysis, moisture migration, and local texture formation [4,11,12]. Therefore, two cheeses with similar average sodium contents may exhibit different microbiological and sensory behavior depending on how uniformly and at what stage the salt is distributed [10].

Reducing NaCl may increase water activity and modify the growth and metabolic activity of starter and non-starter lactic acid bacteria, spoilage organisms, and, under certain conditions, foodborne pathogens [13,14]. It can also alter bacterial autolysis, residual lactose metabolism, enzyme activity, proteolysis, and flavor-compound formation [11,15]. Changes in ionic strength and mineral equilibria affect casein hydration and protein–protein interactions, potentially modifying firmness, cohesiveness, meltability, syneresis, and mouthfeel [16,17,18,19]. These effects are neither linear nor universal: their magnitude depends on cheese composition, pH, moisture, fat content, salting method, starter system, ripening period, and storage conditions [11,20]. Consequently, a level of sodium reduction that is technologically acceptable in one cheese variety may cause pronounced sensory or structural defects and compromise microbial stability in another. To mitigate these effects, complementary natural preservation strategies have been investigated to enhance antioxidant activity, control microbial growth, and extend the shelf life of low-salt cheeses [21,22].

The challenge is further complicated by consumer expectations. Salt contributes directly to salty taste but also suppresses bitterness, enhances characteristic cheese flavors, and modifies aroma release. Partial replacement of NaCl with potassium, calcium, or magnesium salts can preserve some of its physicochemical and antimicrobial functions, but excessive substitution may introduce bitter, metallic, or otherwise unfamiliar sensory notes [23]. Successful reformulation must therefore balance sodium reduction against microbial safety, structural integrity, flavor development, labeling requirements, cost, and consumer acceptance.

Rather than considering salt-reduction techniques as isolated interventions, this review adopts an integrated, cheese-specific perspective. It critically examines conventional approaches, including direct NaCl reduction, partial substitution with mineral salts, flavor enhancement, and modification of manufacturing and ripening conditions, alongside emerging strategies based on controlled salt delivery, improved salt distribution, clean-label ingredients, selected starter and adjunct cultures, and predictive process optimization. The developing roles of artificial intelligence and multi-omics are also considered as tools for relating formulation and processing variables to microbial dynamics, metabolite formation, texture, and sensory performance [24,25]. Attention is given to the transferability of experimental strategies to industrial production while maintaining microbial safety, shelf stability, regulatory compliance, and consumer acceptance. The central premise is that successful sodium reduction requires the multiple technological functions of NaCl to be compensated through coordinated formulation and process controls, rather than by simply removing salt.

Relevant peer-reviewed literature was identified through targeted searches and citation tracking, with priority given to experimental studies quantifying the effects of NaCl reduction or substitution on cheese composition, ripening biochemistry, microbial ecology and safety, texture, and sensory performance. Authoritative regulatory documents were also considered where appropriate. Collectively, the evidence demonstrates that sodium reduction in cheese is not simply a compositional reformulation; it requires a cheese-specific redesign of the preservative, biochemical, structural, and sensory functions normally provided by NaCl.

## 2. Technological Roles of Sodium Chloride in Cheese

Sodium chloride is an indispensable component of cheese manufacture, exerting a major influence on product quality, sensory characteristics, microbial stability, and safety [13,26]. Regardless of the stage or method of addition, NaCl performs several interrelated functions during cheese manufacture and ripening. It promotes whey expulsion and thereby contributes to the control of final moisture content and water activity [21,27]. It also regulates the metabolism and viability of starter cultures, non-starter lactic acid bacteria, and secondary microorganisms involved in the production of volatile flavor compounds during ripening. Furthermore, NaCl modulates enzyme activity and influences protein hydration, ionic strength, and calcium–casein equilibria, thereby affecting cheese structure, firmness, and overall body [9,16,28]. In addition to these technological functions, it contributes directly to saltiness and enhances the perception and balance of characteristic cheese flavor [29].

Salt concentration also influences glycolysis, acid development, proteolysis, and lipolysis during ripening [16,28]. Proteolysis is essential for the development of cheese texture and flavor, but its rate must remain controlled to prevent excessive softening, bitterness, and other quality defects [28]. By binding available water and decreasing α_w_, NaCl restricts the growth of undesirable microorganisms and contributes to cheese safety and shelf-life [5]. During salting, osmotic pressure promotes syneresis and whey expulsion; therefore, insufficient salting may increase moisture retention and alter the distribution of fat and protein in the cheese matrix. Reduced NaCl concentrations may also accelerate lactose metabolism, proteolysis, and lipolysis by increasing microbial and enzymatic activity [21,30]. Consequently, reduced-sodium cheeses may differ substantially from conventionally salted products in moisture content, composition, acidity, texture, flavor development, microbial stability, and ripening behavior [11,13].

The technological significance of NaCl varies considerably among cheese varieties because of differences in composition, moisture content, salting method, ripening conditions, and associated microbiota. Accordingly, sodium content differs substantially both among cheese categories and among products within the same category. Table 1 presents literature-derived sodium intervals together with the corresponding USDA FoodData Central SR Legacy reference values, where available, while distinguishing among analytically measured, label-derived, calculated, and imputed data [31]. These complementary data provide a transparent compositional context without implying that a single value represents the universal sodium content of a given cheese variety. The table is not intended to compare individual sodium-reduction interventions, which are discussed separately because their formulations, analytical parameters, and ripening or storage conditions are not directly comparable. Collectively, the evidence demonstrates that NaCl is not merely a source of saltiness but a multifunctional ingredient whose effects on moisture regulation, texture, ripening biochemistry, flavor development, microbial ecology, and product stability are strongly cheese-specific.

### 2.1. Flavor

Sodium chloride is the principal contributor to salty taste in cheese. Although potassium and calcium salts can also produce saltiness, they are generally less effective than NaCl and may impart undesirable bitter, metallic, or mineral aftertastes, particularly at high substitution levels [47,48,49]. The sensory effect of NaCl extends beyond the perception of saltiness, as it enhances characteristic cheese flavors, improves overall flavor balance, and suppresses the perception of bitterness [48,50].

NaCl also influences flavor development indirectly by regulating starter and non-starter microbial activity, enzyme function, proteolysis, lipolysis, and the formation of volatile compounds during storage and ripening. Appropriately salted cheeses therefore generally exhibit greater flavor intensity and a more balanced sensory profile than unsalted or insufficiently salted cheeses, which may be perceived as bland, watery, or weak in characteristic flavor [49]. Bitterness in cheese is commonly associated with the accumulation of hydrophobic peptides generated during proteolysis [50]. NaCl can affect both the formation and further degradation of these peptides while simultaneously suppressing their sensory perception. Therefore, the relationship between salt concentration and bitterness is complex and depends on cheese variety, salt-in-moisture content, starter and adjunct cultures, proteolytic activity, and ripening conditions [23,51]. Excessive salt may inhibit desirable biochemical reactions, whereas insufficient salt may accelerate proteolysis and contribute to the accumulation of bitter compounds [50].

### 2.2. Texture

Sodium chloride affects cheese texture through its influence on moisture content, protein hydration, ionic strength, mineral equilibria, and biochemical changes during ripening. NaCl modifies the hydration and water-binding capacity of the casein matrix and, consequently, the firmness, elasticity, cohesiveness, and meltability of cheese [5,26]. It also promotes whey expulsion during salting, thereby contributing to the control of final moisture content. Reducing NaCl generally results in greater moisture retention and higher water activity, which may produce a softer cheese matrix and alter its rheological and cooking properties [4,29].

The relationship between NaCl concentration and casein hydration is not linear. Studies using model casein or para-casein systems have indicated that NaCl concentrations of approximately 5–6% (*w*/*w*) may increase protein hydration and solubility under specific pH and temperature conditions [4,29]. This effect is associated with changes in ionic strength and calcium–casein equilibria. Calcium ions act as structural bridges within the casein network because their divalent nature promotes interactions and aggregation among protein molecules [11,18]. Acidification promotes the solubilization of colloidal calcium phosphate and the migration of calcium from the casein matrix to the aqueous phase. Partial exchange of casein-bound calcium with monovalent sodium ions may further increase casein hydration and weaken protein–protein interactions. However, at higher NaCl concentrations, reduced protein hydration and salting-out effects may occur, producing a firmer and more rigid cheese matrix [18].

These mineral and hydration changes are particularly relevant to brined cheeses. As the pH decreases during manufacture and ripening, especially within the approximate range of 5.3–5.1, calcium becomes increasingly solubilized and may migrate from the cheese matrix into the surrounding aqueous phase or brine. Under specific brining conditions, NaCl may promote additional calcium redistribution, increase para-casein hydration, and soften the cheese matrix. In extreme cases, partial solubilization of protein fractions and their migration into the brine may occur, negatively affecting cheese structure and brine stability [48]. Salt reduction can therefore influence handling, cutting, packaging, mastication, and melting behavior, all of which are important quality attributes for manufacturers and consumers. Reduced-salt cheeses are frequently described as softer, weaker, more adhesive, or pasty because of their higher moisture content, increased casein hydration, and accelerated proteolysis. Conversely, cheeses containing excessive NaCl may develop a dry, hard, short, or brittle body because of increased whey expulsion, reduced protein hydration, and inhibition of proteolytic activity. Nevertheless, these effects are cheese-specific and depend on S/M content, pH, calcium concentration, fat and moisture levels, manufacturing conditions, and ripening time [4,29].

The structural consequences of NaCl reduction differ among cheese categories. In hard and semi-hard cheeses, reduced salt-in-moisture may increase moisture retention and accelerate proteolysis during ripening, progressively weakening the casein matrix. Sihufe et al. [52], for example, found that reducing NaCl in Tybo cheese from 1.28 to 0.91 or 0.46 g/100 g increased moisture, casein degradation, and the maturation index and produced a more open microstructure [52]. In Swiss-type cheese, however, Gagnaire et al. (2022) found that a 30% NaCl reduction caused only limited changes in microbial and biochemical characteristics, demonstrating that moderate reduction may be feasible when starter and ripening cultures are appropriately selected [53]. In fresh and high-moisture cheeses, the limited ripening period means that the principal consequences are more directly associated with syneresis, moisture retention, pH, cold-storage microbiology, and short-term texture. Results from Mozzarella-style and Minas fresh cheeses should nevertheless be interpreted cautiously when salt reduction is combined with changes in fat, calcium, or plant-extract concentration because these simultaneous interventions confound the independent effect of NaCl.

### 2.3. Cheese Ripening

Cheese ripening involves a series of interdependent biochemical, physicochemical, and microbiological transformations that generate the characteristic flavor, aroma, texture, and appearance of each cheese variety. As illustrated in Figure 1, the three principal biochemical pathways of cheese ripening are glycolysis, proteolysis, and lipolysis. They are mediated by residual coagulant, indigenous milk enzymes, starter and non-starter microorganisms, secondary microbiota, and their intracellular and extracellular enzymes. Their rates are strongly influenced by pH, temperature, moisture, water activity, and particularly the salt-in-moisture content of the cheese [48].

#### 2.3.1. Microbial Ecology and Metabolic Regulation

Cheese represents a dynamic fermented-food ecosystem in which interacting populations of lactic acid bacteria, yeasts, and fungi transform milk constituents into organic acids, short-chain fatty acids, exopolysaccharides, bioactive peptides, and other metabolites that contribute to the biochemical, sensory, and functional properties of the final product [54]. During cheese manufacture and ripening, the composition and metabolic activity of these microbial communities change in response to pH, water activity, oxygen availability, temperature, nutrient availability, microbial interactions, and salt concentration [55]. Within this dynamic ecosystem, NaCl functions not only as a preservative but also as an ecological selection factor. By decreasing water activity and imposing osmotic stress, it influences which microbial populations persist and which metabolic pathways predominate during ripening. To maintain cellular turgor and osmotic balance, microorganisms must synthesize or accumulate compatible solutes. These adaptive responses require metabolic energy and may consequently restrict microbial growth, modify substrate transport, and redirect metabolic activity under high-salt conditions. However, salt tolerance differs considerably among microbial species and strains. Starter and non-starter lactic acid bacteria, yeasts, molds, and surface-ripening bacteria may therefore respond differently to the same salt-in-moisture ratio. Consequently, NaCl does not uniformly suppress the cheese microbiota; instead, it modifies competitive relationships and selects populations according to their capacity to tolerate and adapt to the physicochemical conditions of the cheese matrix [19,35,56].

Microbial selection also varies spatially and temporally because salt is not distributed uniformly immediately after dry salting or brining. The inward diffusion of salt, accompanied by the outward migration of moisture, creates transient gradients in salt concentration and water activity between the rind and the cheese core. Microorganisms located near the rind may therefore experience earlier and stronger osmotic selection than populations in the cheese interior. Metagenomic evidence from ewe’s-milk cheese demonstrated that salt concentration interacts with ripening temperature to shape microbial communities from the rind to the core [56]. Moreover, microbial succession during cheesemaking is influenced by microorganisms originating from raw milk and by the selective conditions imposed at successive processing and ripening stages [55]. The final cheese microbiota thus reflects interactions among the initial microbial community, spatial salt distribution, water activity, pH, temperature, oxygen availability, and microbial competition.

Reducing the salt-in-moisture ratio relaxes the osmotic selection imposed by NaCl and may increase the relative abundance or metabolic activity of salt-sensitive microorganisms. However, this response is not uniform across microbial groups because starter bacteria, non-starter lactic acid bacteria, yeasts, molds, and surface-ripening bacteria differ in their salt tolerance and metabolic capabilities. McCarthy et al. (2015) demonstrated that salt reduction altered water activity, lactose metabolism, and microbial populations in Cheddar cheese [15]. Similarly, studies involving soft, semi-hard, and surface-ripened cheeses showed that changes in NaCl concentration modified microbial succession and biochemical activity, with responses determined by cheese composition, microbial community structure, and ripening conditions [19,35,57]. These ecological shifts modify the collective metabolic potential of the cheese microbiota, including its capacity for lactose and citrate metabolism, proteolysis, amino-acid catabolism, and the conversion of fatty acids into volatile compounds. NaCl reduction should therefore be interpreted as a cheese-specific reorganization of microbial communities and their metabolic functions, rather than as a uniform increase in microbial activity.

#### 2.3.2. Glycolysis and Organic-Acid Metabolism

The metabolism of residual lactose, lactate, and citrate contributes to acidification and the development of desirable flavor, texture, and rheological properties. Glycolysis (Figure 1) is the conversion of lactose into lactate by lactic acid bacteria [58,59]. Lactose is first hydrolyzed into glucose and galactose, which are metabolized to pyruvate and subsequently reduced mainly to L-lactate [58,60]. This process lowers the curd pH and influences syneresis, texture, microbial stability, and flavor development. The amount of lactose remaining in the curd depends on the cheese variety and manufacturing conditions, including the extent of whey drainage, curd washing, acidification, and salting. During manufacture and early ripening, starter lactic acid bacteria (SLAB) convert most residual lactose into lactate, predominantly L-lactate, depending on the starter species employed [38,58,61]. Residual lactose may subsequently be metabolized by non-starter lactic acid bacteria (NSLAB), while some microorganisms can convert L-lactate into D-lactate [38,61,62]. Excessive accumulation of D-lactate may promote the formation of poorly soluble calcium lactate crystals, which can appear as undesirable white deposits on the surface or within certain cheeses [48]. Citrate metabolism by citrate-positive LAB produces compounds such as diacetyl, acetoin, acetate, and carbon dioxide. Diacetyl and acetoin contribute buttery notes, whereas carbon dioxide can participate in the formation of small openings or eyes in certain Dutch-type cheeses [63,64]. NaCl influences these pathways mainly by regulating microbial growth and metabolic activity [61,62,65]. High salt-in-moisture (S/M) content levels may inhibit starter cultures, delay lactose utilization, reduce acid production, and result in greater residual lactose concentrations and a higher cheese pH. Nevertheless, the inhibitory threshold is cheese- and culture-dependent; therefore, a value of 5% S/M content should not be presented as universally applicable [48].

#### 2.3.3. Proteolysis and Amino-Acid Catabolism

Proteolysis (Figure 1) is one of the most important and complex biochemical processes occurring during cheese ripening [62]. It contributes to texture development through the progressive breakdown of the casein matrix and to flavor development through the formation of peptides and free amino acids (FAAs). These products may contribute directly to taste or act as precursors of volatile compounds. However, uncontrolled proteolysis may cause excessive softening, bitterness, and other sensory defects [61,62,66]. Primary proteolysis is principally mediated by residual coagulant, particularly chymosin, and by indigenous milk proteinases such as plasmin. Chymosin preferentially hydrolyzes α_s1-casein, generating relatively large peptides that are subsequently degraded by proteinases and peptidases from starter and non-starter bacteria [67,68]. This secondary proteolysis produces smaller peptides and FAAs, which contribute to flavor development and provide substrates for further catabolic reactions. NaCl influences proteolysis through its effects on enzyme activity, protein conformation, microbial growth, and bacterial autolysis [69,70]. Increasing the S/M content generally decreases the rate of proteolysis by reducing water activity and limiting the activity of coagulant and microbial enzymes. Reduced-salt and unsalted cheeses may consequently contain greater concentrations of water-soluble nitrogenous compounds, reflecting more extensive casein degradation. However, the effect is not strictly linear and differs among enzymes and casein substrates. Moderate NaCl concentrations may promote the hydrolysis of α_s1-casein by residual coagulant under certain conditions, whereas higher concentrations generally inhibit enzyme–substrate interactions and retard ripening [69]. The hydrolysis of β-casein is particularly sensitive to salt concentration. The effect of pH must also be considered because acidification affects the conformation and accessibility of caseins and modifies the activity of individual proteolytic enzymes. Plasmin preferentially hydrolyzes β-casein and is particularly important in Swiss-type, surface-ripened, and mold-ripened cheeses, in which the pH may increase during ripening [71,72]. It also plays an important role in cheeses manufactured at relatively high curd-cooking temperatures because plasmin is more heat-stable than chymosin and may retain activity when much of the residual coagulant has been inactivated [48].

#### 2.3.4. Lipolysis and Volatile-Compound Formation

Lipolysis involves the enzymatic hydrolysis of milk triacylglycerols and the release of free fatty acids (FFAs). FFAs contribute directly to cheese flavor, particularly when they have short or medium carbon chains, and also serve as precursors of methyl ketones, secondary alcohols, esters, and lactones. Lipolysis is particularly important in blue cheeses and hard Italian-style varieties, in which FFAs and their secondary metabolites make substantial contributions to the characteristic aroma profile. NaCl may influence this pathway directly by modifying lipase activity and indirectly by regulating water activity, microbial growth, substrate accessibility, and the activity of lipolytic microorganisms.

Compared with glycolysis and proteolysis, the effects of sodium reduction on cheese lipolysis remain less extensively investigated and are not consistent among cheese varieties. Katsiari et al. (2000) found that partial substitution of NaCl with KCl did not significantly affect either acid degree values or the concentrations of individual FFAs during Feta cheese ripening, indicating that this substitution did not modify primary lipolysis [16]. Similarly, McCarthy et al. (2015) reported that salt reduction exerted only limited effects on total and individual FFA concentrations in Cheddar-style cheese, whereas changes in fat content had a more pronounced influence [15]. These findings suggest that reducing NaCl does not necessarily accelerate the initial hydrolysis of milk triacylglycerols. The finding of Katsiari et al. is directly supported by the study’s measurements of acid degree values and individual FFAs. By contrast, Murtaza et al. [73] showed that decreasing the NaCl addition from 2.5% to 0.5% of curd weight in buffalo-milk Cheddar increased water activity and proteolysis, decreased hardness, toughness, and crumbliness, increased the concentrations of several volatile flavor compounds, and reduced sensory acceptability. However, the increase in volatile compounds should not be interpreted as direct evidence of enhanced lipolysis because volatile formation may also result from changes in microbial metabolism, proteolysis, amino-acid catabolism, and the secondary conversion of lipid-derived substrates. This distinction is important because salt reduction may alter the aroma profile even when total FFA release remains largely unchanged [73].

Evidence from Greek pasta-filata cheese further demonstrates that free-fatty-acid accumulation and volatile formation do not necessarily proceed in parallel. In Kashkaval of Pindos, Pappa et al. [74] reported greater lipolysis in raw-milk cheese produced without an added starter than in pasteurized-milk cheese manufactured with a commercial starter; esters predominated in mature raw-milk cheese, whereas ketones were more abundant in pasteurized-milk cheese [74]. Pappa et al. [75] similarly found comparable total free-fatty-acid contents in sheep-milk cheese and cheese containing 10% goat milk, despite higher total volatile-compound concentrations in the exclusively sheep-milk cheese at 60 and 90 days [75]. Although these studies did not manipulate NaCl, they demonstrate that microbial ecology and processing conditions must be controlled when assessing the effects of salt reduction on lipolysis and aroma formation.

Collectively, the available evidence does not support a universal relationship between NaCl reduction and increased lipolysis. The response depends on cheese composition, milk source, microbial ecology, lipase activity, moisture content, water activity, and ripening conditions. Moreover, primary lipolysis, defined as the release of FFAs, should be distinguished from the subsequent microbial conversion of FFAs into volatile aroma compounds. Sodium reduction may therefore affect cheese aroma through changes in secondary lipid metabolism without necessarily increasing the overall extent of triacylglycerol hydrolysis.

### 2.4. Water Activity

Water in cheese is not uniformly available for microbial growth or biochemical reactions [13,33]. Water activity (α_w_), which reflects the availability rather than the total amount of water, is therefore a key physicochemical parameter governing microbial activity and biochemical changes during cheese ripening [76]. Although water distribution exists as a continuum, it can be operationally described in three main fractions: (i) strongly bound water associated with proteins and other matrix components; (ii) immobilized or entrapped water retained within the pores and capillaries of the casein network; and (iii) free or mobile water, which can migrate through the cheese matrix and participate more readily in chemical, enzymatic, and microbial processes [13,77]. Water activity (α_w_) does not represent the total moisture content of cheese; rather, it expresses the thermodynamic availability of water for microbial growth and biochemical reactions [13,48].

During dry salting or brining, NaCl diffuses into the cheese matrix, while water and some soluble components migrate in the opposite direction because of the resulting osmotic gradient [13]. This exchange promotes whey expulsion, reduces the amount of mobile water, and increases the concentration of dry matter. Once dissolved in the cheese serum, sodium and chloride ions lower the chemical potential and vapor pressure of water, thereby decreasing α_w_. Consequently, S/M content is generally a more useful indicator of water availability than the total salt concentration of the cheese.

The effect of NaCl on protein hydration is more complex and depends on salt concentration, pH, calcium distribution, protein composition, temperature, and ripening conditions. Changes in ionic strength may alter casein–casein interactions and calcium–casein equilibria, thereby modifying the amount and mobility of water retained within the protein network. Moderate salt concentrations may increase casein hydration under certain conditions, whereas higher ionic strength can decrease hydration and promote a more compact protein structure. Therefore, the effect of salting on moisture distribution cannot be explained solely by the water-binding capacity of NaCl but must be considered in relation to the physicochemical properties of the entire cheese matrix [48].

### 2.5. Microbial Ecology and Safety Under Reduced-Salt Conditions

Sodium chloride contributes to cheese preservation primarily by decreasing water activity (α_w_) and imposing osmotic stress that restricts microbial growth and metabolic activity. However, its antimicrobial effect is predominantly bacteriostatic at the concentrations normally encountered in cheese and operates together with pH, organic acids, redox potential, moisture, storage temperature, packaging, and microbial competition. Consequently, lowering the salt-in-moisture ratio may weaken one component of this hurdle system, but it does not necessarily result in equivalent responses among starter bacteria, non-starter lactic acid bacteria, spoilage microorganisms, and foodborne pathogens.

Experimental evidence confirms that the microbiological consequences of salt reduction are cheese-specific. Dugat-Bony et al. [35] reduced the NaCl content of a model surface-ripened cheese from 1.8% to 1.3% [35]. The reduced-salt treatment decreased the development of *Debaryomyces hansenii* while promoting *Hafnia alvei* and the spoilage bacterium *Pseudomonas fragi*. These ecological changes were accompanied by differences in proteolysis, volatile-compound formation, and biogenic-amine accumulation. This evidence demonstrates that even a moderate reduction in NaCl can reorganize microbial interactions and metabolic functions, particularly in cheeses containing complex surface microbiota.

The response of starter and ripening microorganisms is also dependent on the organisms and salt-replacement strategy employed. Simov and Ivanov [78] reported that partial replacement of NaCl with KCl or KH_2_PO_4_ in Kashkaval cheese did not significantly affect the final counts *of Lactobacillus delbrueckii* subsp. *bulgaricus* or the measured indices of proteolysis [78]. These findings indicate that an appropriately formulated mineral-salt mixture may preserve selected fermentation and ripening functions. Nevertheless, measurements of starter survival and proteolysis do not demonstrate pathogen control; microbiological safety must be assessed separately using pathogen-specific evidence.

Available challenge studies suggest that the safety consequences of sodium reduction are strongly dependent on the cheese matrix and contamination conditions. Hystead et al. [79] found that sodium reductions of up to 50%, achieved with or without partial replacement by KCl, did not significantly alter the survival of Listeria monocytogenes in Cheddar cheese [79]. However, the pathogen survived more effectively when inoculation occurred at a later manufacturing stage, demonstrating the importance of preventing post-process contamination. In Edam cheese, Luzzi et al. (2021) similarly reported that direct sodium reduction of approximately 30%, or a reduction of approximately 50% using a potassium-containing salt replacer, did not produce significant changes in the bacterial community or in the behavior of an inoculated *Listeria innocua* surrogate during six weeks of storage [80]. These results are encouraging but should not be extrapolated indiscriminately to cheeses with different pH, moisture content, ripening microbiota, processing conditions, or shelf-life.

Evidence from Greek Kashkaval production also illustrates the importance of processing controls. Samelis et al. [81] demonstrated that curd heating and stretching during pasta-filata manufacture produced substantial reductions in coagulase-positive staphylococci and coliform bacteria and minimized the presence of *Listeria* spp. [81]. Nevertheless, the lethality of the stretching step depends on the time–temperature conditions and microbial load and cannot compensate for contaminated raw milk, inadequate acidification, or post-processing contamination.

Food safety under reduced-salt conditions should consequently be supported by good agricultural practices and good manufacturing practices throughout the production chain [12,66,82]. At farm level, relevant controls include herd-health and mastitis management, hygienic milking, prevention of fecal contamination, use of potable water, rapid milk cooling, and microbiological monitoring of raw milk. During cheese manufacture, critical measures include validated milk heat treatment where applicable, controlled starter activity and acidification, hygienic handling, effective cleaning and disinfection, microbiological management of brines, prevention of cross-contamination, environmental monitoring for *Listeria* spp., appropriate packaging, and continuous maintenance of the cold chain.

Reduced-sodium formulations should be validated throughout their intended shelf-life by combining physicochemical measurements particularly salt-in-moisture ratio, water activity, pH, moisture, and organic-acid content with microbial enumeration and pathogen-specific challenge testing [4,48]. Testing should include both recommended storage conditions and reasonably foreseeable temperature abuse. Where NaCl reduction weakens microbial stability, additional hurdles such as optimized acidification, selected protective cultures, refrigeration, modified-atmosphere packaging, or validated food-grade antimicrobial compounds may be required. Current evidence does not support a universal microbiologically safe level of NaCl reduction; safety limits must instead be established for each formulation, process, cheese matrix, packaging system, and storage condition.

### 2.6. Shelf-Life and Safety

Sodium chloride is an important preservation hurdle in cheese because it limits microbial growth and regulates the activity of starter, non-starter, spoilage, and potentially pathogenic microorganisms [47]. Its preservative action is primarily associated with the reduction in α_w_ and the development of an osmotic gradient between microbial cells and the surrounding aqueous phase [13,18]. This osmotic stress causes water loss from microbial cells, disrupts membrane function and nutrient transport, and may inhibit cellular metabolism. At concentrations normally encountered in cheese, the effect of NaCl is predominantly bacteriostatic rather than bactericidal [13,14,24].

The antimicrobial effect of NaCl operates in combination with other intrinsic and extrinsic factors, including pH, organic acid concentration, redox potential, storage temperature, competing microbiota, packaging conditions, and moisture content. This combination constitutes a hurdle system that controls microbial survival and growth during ripening and storage. Salt therefore contributes to safety and shelf-life, but it cannot compensate for inadequate hygiene, insufficient acidification, temperature abuse, or post-processing contamination [13,20].

NaCl also affects desirable cheese microbiota. Increasing salt concentration may delay starter growth, acidification, and lactose metabolism, whereas lower concentrations generally permit greater starter activity [19,55,56]. However, salt tolerance varies substantially among microbial species and strains [56,83]. Non-starter lactic acid bacteria are often more salt-tolerant than starter cultures, while some yeasts, molds, and halotolerant bacteria may remain active at relatively high S/M levels. Consequently, no universal NaCl concentration such as 2% can be considered inhibitory to all bacteria [35,56,83].

Each cheese variety has a characteristic S/M ratio that balances microbial control with desirable starter activity and ripening. Reducing S/M ratio generally increases α_w_ and may promote the growth of undesirable microorganisms, accelerate enzymatic reactions, and shorten shelf-life [13,33,76]. It may also alter the relative populations of starter lactic acid bacteria (SLAB) and non-starter lactic acid bacteria (NSLAB), with subsequent effects on pH development, glycolysis, proteolysis, lipolysis, flavor formation, and texture [50]. Nevertheless, sodium reduction does not automatically make a cheese unsafe; the resulting risk depends on the extent of reduction, cheese composition, manufacturing process, storage conditions, and the presence of complementary preservation hurdles that can even be sourced from food-grade by-products [14,15,19,24,76,84].

Scientific evidence indicates that the microbiological consequences of NaCl reduction are both cheese- and microorganism-specific [15,19,56,57]. Reducing the salt-in-moisture ratio generally increases water activity, but the resulting microbial response is not uniform. Greater starter activity may accelerate acidification and thereby strengthen one preservation hurdle, whereas enhanced growth of non-starter bacteria, spoilage microorganisms, or salt-tolerant pathogens may shorten shelf-life or compromise safety. In particular, Wemmenhove et al. [33] demonstrated that NaCl and moisture jointly determine water activity and the predicted inhibition of *Listeria monocytogenes*, showing that microbiological stability cannot be inferred from the nominal percentage of sodium reduction alone. Instead, it results from the combined effects of water activity, pH, organic-acid concentration, moisture content, storage temperature, microbial competition, and packaging conditions [33].

Reducing the antimicrobial contribution of NaCl consequently increases the relative importance of controlling the initial microbial load and strengthening complementary preservation hurdles. Evidence showing that raw-milk microbiota influences microbial succession throughout cheesemaking provides a scientific basis for applying Good Agricultural Practices, particularly good dairy farming practices, at the primary-production level and Good Manufacturing and Hygiene Practices during cheese manufacture [55]. Additional measures, including controlled acidification, selected protective cultures, refrigeration, modified-atmosphere packaging, and food-grade antimicrobial ingredients, may partially compensate for the weakened NaCl hurdle [21,84]. Nevertheless, their effectiveness remains dependent on the cheese matrix and processing conditions. Reduced-sodium formulations should therefore be validated through microbial enumeration, pathogen-specific challenge testing, and shelf-life studies conducted under both recommended and reasonably foreseeable storage conditions. Collectively, the available evidence indicates that the relevant safety endpoint is not a predetermined percentage of NaCl reduction, but the experimentally demonstrated microbial stability of the complete cheese system throughout its intended shelf-life.

## 3. Techniques for Sodium Chloride Reduction

Sodium-reduction strategies in cheese can be broadly classified into formulation and substitution, salt-delivery and sensory-design, and process and safety-optimization approaches. Formulation strategies include direct NaCl reduction, partial replacement with alternative mineral salts, and the incorporation of functional ingredients that support flavor or microbial stability [4,13]. Salt-delivery and sensory-design approaches include flavor enhancement, modification of salt-crystal size and morphology, and spatially heterogeneous salt distribution to increase perceived saltiness [13,18]. Process and safety-optimization strategies involve controlling brining and salting conditions, modifying cheesemaking operations, and applying complementary technologies such as precision salting, protective cultures, packaging, and validated hurdle systems. Simply lowering NaCl may adversely affect flavor, texture, ripening, microbial stability, and shelf-life [49]. Potassium chloride (KCl) is the most widely studied substitute because it provides a salty taste and partially reproduces the technological functions of NaCl [4]. Nevertheless, high substitution levels may impart bitter or metallic notes and alter microbial and biochemical processes [50,57]. Consequently, effective sodium reduction generally requires a cheese-specific combination of complementary strategies. The principal approaches for sodium reduction in cheese are summarized in Figure 2 and discussed in the following subsections.

### 3.1. Direct Reduction in NaCl

Direct NaCl reduction involves decreasing the amount of sodium chloride added during cheesemaking without replacing it with another salt. Although this is the simplest sodium-reduction strategy, lowering NaCl changes the salt-in-moisture ratio, water activity, ionic balance, enzyme activity, and microbial ecology of cheese, potentially affecting moisture retention, texture, proteolysis, flavour development, safety, and shelf-life [13,85]. These interconnected physicochemical, microbiological, and technological effects are summarized in Figure 3. The feasibility of this strategy therefore depends on the cheese variety, initial salt content, salting method, manufacturing conditions, and ripening system [13,86].

Among studies evaluating this approach, Soares et al. [87] examined São João cheese manufactured using curd dry-salting levels of 4% NaCl (control), 3%, 2%, 1%, and 0% (*w*/*w*), corresponding to NaCl reductions of 25–100%. The cheeses were ripened at 11 °C for 40 days and evaluated for physicochemical, microbiological, and sensory characteristics. At equivalent ripening times, decreasing NaCl was associated with lower moisture, ash, and sodium contents and a higher protein content, whereas pH, acidity, fat content, and the microbiological parameters evaluated did not differ significantly among treatments. Triangle testing detected differences from the control at NaCl levels of 2% or lower but not at 3% NaCl. Under the conditions examined, a 25% reduction therefore produced cheese that was not distinguishable from the control by regular consumers and was considered potentially feasible for industrial production [87].

Whereas Soares et al. [87] evaluated NaCl reduction as the principal formulation change, Henneberry et al. [39] examined the combined effects of reducing salt from approximately 1.7% to 1.0% and fat from approximately 22% to 11% in Mozzarella-style cheese. Simultaneous salt and fat reduction increased protein and moisture contents but decreased moisture-in-non-fat substances, fat-in-dry matter, and salt-in-moisture. These compositional changes were accompanied by lower water-binding capacity and primary proteolysis, as well as greater hardness and chewiness in the unheated cheese. The reduced-fat, reduced-salt cheese also exhibited lower flowability and greater resistance to extension after heating. Although lowering calcium partially mitigated these effects, it did not fully restore the functional properties of the full-fat, full-salt control. Because fat and salt were reduced simultaneously, the observed changes cannot be attributed exclusively to NaCl reduction; nevertheless, the findings demonstrate the technological challenges associated with combined salt and fat reformulation in Mozzarella-style cheese [39].

Extending this combined reformulation approach to a long-ripened cheese, McCarthy et al. [15] evaluated the independent and interactive effects of reducing salt and fat by 30% and 50% in Cheddar-style cheese during 270 days of ripening at 8 °C. Reducing salt from 1.9% to 1.2% or 0.9% increased moisture, lactic acid, the lactic acid-to-protein ratio, and water activity, while decreasing residual lactose and starter-culture die-off. Reducing fat from 33% to 22% or 16% increased moisture, protein, and lactic acid but decreased salt-in-moisture, moisture-in-non-fat substances, and the lactic acid-to-protein ratio. Lactose content, pH, and water activity were also influenced by interactions between fat and salt levels. During ripening, pH decreased in full-fat, full-salt cheese, increased in half-fat, reduced-salt and half-fat, half-salt cheeses, and remained relatively stable in the other formulations. These findings demonstrate that salt and fat jointly influence cheese composition, lactose metabolism, starter-culture behaviour, and ripening; consequently, the observed effects cannot be attributed exclusively to NaCl reduction [15].

Highlighting the strong dependence of salt-reduction outcomes on cheese type and ripening microbiota, Dugat-Bony et al. [35] compared a control soft surface-ripened cheese containing 1.8% NaCl with a reduced-salt cheese containing 1.3% NaCl, corresponding to an approximately 28% reduction. The cheeses were sampled weekly during 27 days of ripening. Lowering NaCl disrupted the microbial balance, resulting in reduced development of the yeast *Debaryomyces hansenii* and increased growth of the Gram-negative bacterium Hafnia alvei. These microbial changes were accompanied by altered proteolytic kinetics, volatile-aroma profiles, and biogenic-amine production. Moreover, the spoilage microorganism *Pseudomonas fragi* reached significantly higher populations in the reduced-salt cheese. These findings demonstrate that even moderate NaCl reduction can substantially affect the microbial ecology and biochemical development of surface-ripened cheese, emphasising the need for cheese-specific microbiological and safety validation [35].

Baptista et al. [88] evaluated the effects of reducing NaCl by 25% and 50% on proteolysis, peptide composition, firmness, and sensory acceptance in Prato cheese. Neither reduction level markedly affected proteolysis nor resulted in the formation of known bitter-tasting peptides. Cheese containing 25% less NaCl exhibited a peptide profile, firmness, and sensory acceptance comparable to those of the control. In contrast, a 50% reduction produced a less firm cheese with lower consumer acceptance. These findings indicate that a 25% direct reduction in NaCl may be feasible in Prato cheese, whereas greater reductions are more likely to impair textural and sensory quality [88].

Fosberg and Joyner [40] investigated the effects of NaCl reduction on the rheological, tribological, and sensory properties of cottage cheese cream dressing. Formulations containing 2.20%, 1.48%, or 0.73% NaCl were prepared at pH values of 4.5, 5.0, and 5.5 and evaluated at different temperatures and storage times. All formulations exhibited shear-thinning, frequency-dependent, and predominantly viscoelastic-solid behaviour. The effects of pH on rheological and tribological properties were more pronounced than those of NaCl concentration, while the measured parameters generally increased during storage. Sensory evaluation, conducted on the 2.20% and 0.73% NaCl formulations at pH 5.0, indicated that the reduced-salt dressing had attributes similar to those of the full-salt formulation. These findings suggest that substantial NaCl reduction may be feasible in cottage cheese cream dressing under controlled pH conditions; however, the results apply specifically to the dressing and should not be generalised to the complete cottage cheese product [40].

Extending the assessment of direct salt reduction to a ripened semihard cheese, Sihufe et al. [52] examined Tybo cheeses containing 1.28 g NaCl/100 g (control), 0.91 g/100 g (30% reduction), or 0.46 g/100 g (65% reduction) during 40 days of ripening. Reducing the NaCl content significantly increased moisture, the maturation index, α_s1_-I-casein degradation, and the pH 4.6-soluble fraction, while decreasing the proportion of intact caseins. Salt reduction also lowered the measured rheological parameters and produced a more open microstructure, with larger voids than in the control cheese. Although the overall flavour and texture profiles remained broadly similar among treatments, perceived saltiness, acidity, and overall flavour persistence decreased significantly as the salt content declined. Overall, the 30% NaCl reduction produced a cheese that retained most of the characteristics of conventional Tybo cheese, whereas the 65% reduction caused more pronounced biochemical, structural, and sensory changes [40].

The feasibility of moderate direct salt reduction was further supported by Gagnaire et al. [53] who investigated the combined effects of NaCl concentration and starter-culture selection on the ripening of Swiss-type cheese. Initially, the proteolytic activities of three *Lactobacillus helveticus* strains toward caseins were evaluated in vitro at six NaCl concentrations ranging from 0 to 4.5% and at pH 7.5 and 5.2. Casein hydrolysis was generally similar or greater at pH 5.2 than at pH 7.5 but declined at the highest NaCl concentrations. In the experimental cheeses, the selected *L. helveticus* strains produced 1.5–2.4-fold greater proteolysis than cheeses manufactured without *L. helveticus*, demonstrating that starter selection had a greater influence on proteolysis than moderate salt reduction. Cheeses containing 30% less NaCl exhibited proteolysis and microbial and biochemical characteristics comparable to those of the regular-salt controls. By contrast, increasing the NaCl concentration 2.5-fold slightly reduced the non-protein nitrogen fraction and inhibited the growth and metabolic activity of *Propionibacterium freudenreichii*. Salt reduction caused only small decreases in extrusion and oiling-off properties, whereas flowability and stretchability remained unaffected. These findings indicate that a 30% NaCl reduction is technically feasible in Swiss-type cheese and that appropriate starter and ripening-culture selection may help compensate for changes caused by salt modification. However, because no sensory evaluation was performed, the effect of the reduced NaCl content on perceived saltiness and consumer acceptance remains uncertain [53]. More recently, Yaşar et al. [89] reported that reducing the NaCl concentration from 8% to 4% in the salting formulation of Kashar cheese enhanced proteolysis and produced cheese with lower hardness and greater meltability. However, the conventionally salted cheese remained preferable in terms of flavour, indicating that technological improvements associated with direct salt reduction do not necessarily ensure equivalent sensory acceptance [89].

Overall, the available evidence indicates that direct NaCl reductions of approximately 25–30% are most consistently achieved without substantial impairment of cheese quality, as demonstrated in São João, Prato, Tybo and Swiss-type cheeses by Soares et al., Baptista et al., Sihufe et al. and Gagnaire et al., respectively [52,53,87,88]. Greater reductions may be feasible in specific cheese matrices but are more likely to modify moisture retention, proteolysis, texture, meltability and sensory acceptance [86]. Moreover, the microbial perturbations reported by Dugat-Bony et al. [35] demonstrate that even moderate NaCl reduction can compromise ecological stability in surface-ripened cheeses [35]. Direct reduction must therefore be validated separately for each cheese variety throughout ripening and shelf life, with particular attention to microbial safety and sensory quality. Where the desired sodium reduction cannot be achieved by lowering NaCl alone, partial replacement with mineral salts or complementary reformulation strategies may be required.

### 3.2. Direct Substitution of NaCl with Mineral Chloride Salts

Partial substitution of sodium chloride (NaCl) with mineral salts is among the most extensively studied strategies for reducing sodium in cheese. Potassium chloride (KCl) is used most frequently because it can partly reproduce the ionic, preservative, and salty-taste functions of NaCl [4,18,49]. Moreover, replacing part of the sodium with potassium may improve the dietary sodium-to-potassium balance. Nevertheless, the bitter and metallic aftertaste associated with KCl often limits the acceptable replacement level [50]. Divalent salts, particularly magnesium chloride (MgCl_2_) and calcium chloride (CaCl_2_), may also be used, although their interactions with caseins can alter acidity, protein hydration, texture, enzyme activity, and microbial growth. The effectiveness of mineral-salt substitution therefore depends on the cheese variety, initial NaCl content, substitution level, salting method, ripening conditions, and final salt-in-moisture ratio (S/M) [13,49,90].

Within the European Union, potassium chloride (KCl), calcium chloride (CaCl_2_), and magnesium chloride (MgCl_2_) correspond to E 508, E 509, and E 511, respectively, and their identity and purity specifications are established by Commission Regulation (EU) No 231/2012 [91]. However, the assignment of an E-number does not imply unrestricted authorization for use in every cheese variety. Regulation (EC) No 1333/2008 specifies that substances used primarily to impart taste or for nutritional purposes, including salt replacers, are not regarded as food additives solely on the basis of that use [92]. When these mineral salts perform a technological additive function, their authorization and conditions of use must be assessed for the relevant dairy-product category. Additional restrictions may apply to cheeses registered under protected designation of origin (PDO) or protected geographical indication (PGI) schemes. Consequently, the regulatory suitability of each mineral salt must be evaluated according to its intended function, concentration, cheese category, and applicable product specification.

Among the early studies Katsiari et al. [46] evaluated partial NaCl replacement with KCl in Kefalograviera cheese using five cheesemaking trials in which split curd lots were subjected to brine and dry salting with NaCl alone or NaCl/KCl mixtures at ratios of 3:1 and 1:1. No significant differences were detected among treatments in composition, pH, water activity, instrumental texture, or the sensory attributes evaluated. The 1:1 mixture reduced sodium content by approximately 50% and decreased the molar Na:K ratio from 35.3 in the control to 1.4, whereas the 3:1 mixture produced a ratio of 4.2. Under the conditions examined, partial KCl replacement therefore achieved substantial sodium reduction without measurable deterioration in the physicochemical, textural, or sensory quality of Kefalograviera cheese [46]. Likewise, examining the biochemical consequences of KCl substitution, Katsiari et al. [16] evaluated lipolysis in Feta cheese manufactured using NaCl alone or NaCl/KCl mixtures at ratios of 3:1 and 1:1 during both dry salting and brining, corresponding to KCl replacement levels of 25% and 50%, respectively. Lipolysis was monitored for up to 240 days using the acid degree value method, while individual free fatty acids were analysed via gas chromatography at selected ripening stages. Neither acid degree values nor the qualitative or quantitative profiles of individual free fatty acids differed significantly among treatments. These results indicate that replacing up to 50% of NaCl with KCl did not measurably affect lipolysis under the conditions examined. However, because the study focused specifically on lipolysis, its findings do not independently establish sensory or microbiological equivalence [16].

Extending the assessment beyond lipolysis to microbial viability and bioactive properties, Gandhi et al. [93] investigated NaCl reduction and partial substitution with KCl in Akawi cheese using brines containing 10% NaCl, 7.5% NaCl, 7.5% total salt as a 1:1 NaCl/KCl mixture, 5% NaCl, or 5% total salt as a 1:1 NaCl/KCl mixture [93]. Lower-salt brines improved probiotic survival after 30 days of storage. The 7.5% NaCl/KCl brine also increased the concentrations of phenylalanine, tryptophan, valine, and leucine, whereas angiotensin-converting enzyme-inhibitory and antioxidant activities remained unaffected. Cheese extracts from the reduced-NaCl treatment also affected normal and cancer-derived colon cell lines; however, these in vitro findings cannot be interpreted as evidence of clinical health benefits [93].

Riad et al. [90] evaluated the partial substitution of NaCl with KCl or MgCl_2_ at different ratios in Kareish cheese. Increasing substitution decreased total solids but increased cheese yield. The substituted cheeses also exhibited lower hardness, gumminess, and chewiness and greater cohesiveness, adhesiveness, and springiness than the NaCl control. Overall, the mineral-salt mixtures produced a softer and smoother cheese, although the magnitude of these effects depended on the NaCl-to-replacer ratio [90]. Carmi and Benjamin [94] examined NaCl reductions of 30% and 50%, with or without salt-replacer blends, in Tzfat cheese. A 50% reduction markedly decreased perceived saltiness, softened the texture, and increased proteolysis. Cheeses containing 30% or 50% KCl also exhibited lower hardness and springiness, although the product containing 30% KCl had the sensory profile most similar to that of the control [94]. Abd El-Aziz et al. [95] investigated Caerphilly cheese dry-salted at 1% of the wet-curd weight and subsequently immersed in either a 25% NaCl brine or 25% total-salt brines containing NaCl/KCl ratios of 90:10, 80:20, and 70:30. Cheese produced using the 70:30 NaCl/KCl mixture achieved higher sensory scores, both when fresh and during storage, than the NaCl control. However, the control showed a slightly higher cheese yield and total solids content, as well as lower total bacterial, lactic acid bacterial, mold, and yeast counts. Despite these differences, the authors concluded that replacing 10–30% of NaCl with KCl did not cause marked overall deviations from conventionally salted Caerphilly cheese. These findings indicate that KCl replacement levels of up to 30% may be technologically and sensorially feasible, although their effects on microbial populations should be carefully monitored [95].

Golin Bueno Costa et al. [96] examined the replacement of NaCl with 25% or 50% KCl in Minas Padrão cheese. Sodium concentrations decreased from 608.67 mg/100 g in the control to 210.05–369.12 mg/100 g in the substituted cheeses. NaCl replacement did not substantially affect pH, proteolysis, or most physicochemical characteristics. However, hardness and chewiness increased, possibly because the lower pH promoted syneresis and reduced moisture retention. Changes in the proportions of short-chain, medium-chain, and polyunsaturated fatty acids were also reported. Consumer testing indicated that 25% KCl replacement was acceptable [96]. Dugat-Bony et al. (2019) evaluated a 20% reduction in NaCl, combined with KCl substitution, in Camembert-type and Reblochon-type cheeses [57]. Physicochemical effects were more pronounced in the soft cheese than in the semi-hard cheese. In the semi-hard cheese, reducing NaCl favored the growth of spoilage-associated Pseudomonas and increased proteolysis and lipolysis. In the soft cheese, NaCl concentration had a limited effect on the growth of *Yarrowia lipolytica* but substantially influenced proteolysis. Reduced-salt treatments in both cheese types exhibited greater odor and taste intensity, although sensory differentiation according to NaCl content was clearer in the soft cheese. These findings demonstrate that the effects of salt replacement are strongly dependent on the cheese matrix and associated microbiota [57].

Zonoubi and Goli [97] examined the complete replacement of NaCl with KCl, MgCl_2_, or CaCl_2_ in the brine of sodium-free ultrafiltered Feta-type cheese. All chloride salts inhibited microbial growth to different degrees. CaCl_2_ exhibited an inhibitory effect similar to NaCl, whereas KCl and MgCl_2_ were progressively less inhibitory. NaCl produced the highest initial hardness and syneresis, whereas MgCl_2_ produced the lowest values after 60 days. Among the substituted treatments, only KCl-brined cheese achieved overall acceptance comparable to that of the NaCl-brined control, indicating that technological functionality alone does not guarantee sensory equivalence [97]. Miocinovic et al. [98] investigated the partial and complete replacement of NaCl with KCl in Serbian white-brined cheese (Homoljski Sir) during 60 days of ripening. The experimental cheeses were produced by replacing 50, 75, or 100% of NaCl with KCl, while the control contained only NaCl. Neither partial nor complete replacement significantly affected the basic composition, proteolysis, or textural properties of the cheese. Sodium content decreased progressively as KCl replacement increased, although replacing 75% of NaCl was still insufficient for the cheese to meet the low-sodium criterion applied in the study because of its high initial salt content. Consumer responses varied substantially among demographic groups. Highly educated female consumers showed low acceptance of all KCl-containing cheeses, whereas male consumers disliked only the cheese in which NaCl was completely replaced. Nevertheless, nearly 80% of all participants moderately to strongly liked the cheese containing 75% KCl replacement. These findings indicate that replacing 50–75% of NaCl with KCl may be technologically feasible and acceptable to a substantial proportion of consumers. However, consumer segmentation, the final measured sodium content, and potential KCl-related bitter or metallic notes should be considered when developing reduced-sodium white-brined cheese [98].

AlQtaishat et al. [99] investigated KCl and MgCl_2_ formulations treated to reduce their inherent bitterness in white-brined cheese. Partial substitution of NaCl with treated KCl, MgCl_2_, CaCl_2_, or their mixtures achieved sodium reductions of up to 80% and generally increased cheese acidity. The formulation containing 50% treated KCl received higher scores for overall liking and saltiness liking than the NaCl control. These findings indicate that the sensory limitations of mineral-salt replacers can be partly overcome through bitterness-reduction treatments and appropriate salt blending [99]. Lavasani [100] investigated the effects of replacing NaCl with KCl in Iranian ultrafiltered white cheese. The treatments contained 3% NaCl (control), 1.5% NaCl and 1.5% KCl, 1% NaCl and 2% KCl, or 0.75% NaCl and 2.25% KCl. Partial replacement did not substantially affect moisture, dry matter, fat, proteolysis, or the free-fatty-acid profile, and enabled a measured sodium reduction of up to 50%. Nevertheless, increasing KCl concentration progressively reduced sensory acceptance and produced cheeses that were more fracturable and less firm, with changes also observed in their aroma profiles [100].

Yaşar et al. [89] investigated direct NaCl reduction and partial replacement with KCl in Kashar cheese during 60 days of ripening. Four salting formulations were evaluated: 8% NaCl (control), 4% NaCl, 4% NaCl plus 4% KCl, and 2% NaCl plus 6% KCl. NaCl reduction or replacement did not significantly affect pH, moisture, or fat content but influenced salt, protein, and ash contents. Proteolysis was enhanced in the cheese containing 4% NaCl and in that with the highest KCl replacement, while the latter also exhibited the highest concentration of total free amino acids. Reduced-sodium cheeses generally showed lower hardness and greater meltability than the control. Although the control received the highest flavor scores, the texture of the reduced-sodium cheese was preferred. These findings demonstrate that partial KCl replacement can reduce sodium and improve certain textural and melting properties of Kashar cheese; however, maintaining flavor acceptability remains an important limitation, particularly at high replacement levels [89].

Overall, the available evidence identifies KCl as the most practical individual mineral substitute for NaCl in cheese. Replacement levels of approximately 25–50% most consistently achieve meaningful sodium reduction while maintaining acceptable physicochemical, microbiological, and sensory properties [94,95,96,100]. Higher replacement levels of 50–75% may also be technologically feasible in selected cheese varieties, particularly when KCl bitterness is mitigated through appropriate treatment or formulation [89,98,99]. However, recent findings in Kashar cheese indicate that the softer texture, greater meltability, and enhanced proteolysis obtained at higher replacement levels may be accompanied by lower flavor acceptance compared with conventionally salted cheese [89]. Complete NaCl replacement remains less reliable because it may reduce perceived saltiness, intensify bitter or metallic notes, and alter texture or microbial inhibition [97,98]. CaCl_2_ and MgCl_2_ may provide additional technological functionality, although their strong interactions with caseins and potential sensory effects generally limit their use as direct substitutes. These limitations have encouraged the development of multi-component mineral-based salt blends intended to provide a more balanced combination of saltiness, technological functionality, microbial stability, and sensory acceptance. Such formulated salt replacers are discussed in the following section.

### 3.3. Mineral-Based Salt Blends and Emulsifying Salt Reformulation

Mineral-based salt blends combine two or more sodium-reduction components to achieve a more favorable balance among saltiness, technological functionality, mineral composition, and sensory acceptance that can generally be obtained using a single NaCl substitute. These formulations may contain NaCl, KCl, calcium salts, and bitterness-masking agents. In processed cheese, sodium reduction may additionally be achieved by replacing sodium-based emulsifying salts with potassium-based citrate or phosphate salts. This distinction is important because emulsifying salts regulate calcium sequestration, casein dispersion, pH, fat emulsification, meltability, and matrix stability and can contribute substantially to the total sodium content of processed cheese.

Luzzi et al. [80] investigated the microbial ecology of reduced-sodium Edam cheese manufactured either through direct sodium reduction or partial substitution with a potassium-containing mineral-salt compound. Microbial diversity was monitored during manufacture and six weeks of ripening using 16S rDNA amplicon sequencing, together with a challenge test employing *Listeria innocua* as a surrogate for *L. monocytogenes*. Neither sodium-reduction strategy significantly altered the overall microbial composition. Starter bacteria remained dominant, and no increased growth of spoilage-associated or potentially pathogenic bacteria was detected. These results support the microbiological feasibility of mineral-salt substitution in Edam cheese under the conditions examined, although they should not be interpreted as evidence of equivalent safety across other cheese varieties or storage conditions [80].

Lisak Jakopović et al. [24] partially replaced NaCl with calcium citrate or calcium lactate at levels of 25% and 50% in the brine of white-brined cheese. Replacement at 25% generally maintained acceptable physicochemical, textural, and sensory properties, with the formulation containing 25% calcium citrate receiving the highest sensory scores among the reduced-sodium treatments. At 50% replacement, the cheeses developed a softer texture and a gritty mouthfeel. These findings suggest that calcium-based non-chloride salts may contribute to sodium reduction, but their limited solubility and effects on protein–mineral interactions restrict the acceptable replacement level [24].

Kurćubić et al. [101] examined a commercial Na/K mineral-salt combination (COMB) as a substitute for 25%, 50%, 75%, or 100% of NaCl in Serbian white soft-brined cheese. Replacement did not significantly affect ash content, pH, or water activity, although the reformulated cheeses exhibited a slightly firmer and more compact microstructure. Sodium content decreased progressively with increasing COMB replacement, reaching 334.80 ± 24.60 mg/100 g in the fully substituted cheese. Under the criteria applied by the authors, this formulation qualified for a “reduced sodium” claim but not as a “low-sodium” product. Overall acceptance did not differ significantly from the control at replacement levels of up to 75%. Complete replacement, however, reduced taste and overall-acceptance scores because of greater bitterness and lower perceived saltiness. Thus, substantial NaCl replacement may be achievable using appropriately designed Na/K blends, whereas complete substitution remains sensorially challenging [101].

A related but technologically distinct strategy involves reformulating the emulsifying-salt system of processed cheese. Chavhan et al. [44] produced low-sodium processed Mozzarella cheese through partial replacement of NaCl with KCl and replacement of sodium-based emulsifying salts with potassium citrate and dipotassium phosphate. Response-surface optimization identified a formulation containing 30.96% KCl and 2% potassium-based emulsifying salts as optimal in terms of sensory quality, hardness, meltability, and stretchability. The conventional- and reduced-sodium cheeses contained 905 and 260 mg sodium/100 g, respectively, corresponding to a 71.27% reduction in measured sodium content. Importantly, these values refer to sodium rather than NaCl. The optimized formulation therefore achieved a substantial sodium reduction without significant deterioration of the evaluated sensory, textural, or functional properties [44].

Nogueira et al. [43] evaluated the partial replacement of a sodium-based commercial polyphosphate blend with monopotassium phosphate in spreadable processed cheese. The different emulsifying-salt systems did not significantly affect the fat or moisture content. Replacement at 25% improved sensory quality, whereas replacement at up to 50% did not cause marked adverse changes in the physicochemical or sensory properties. The 50% replacement reduced measured sodium content by approximately 29%, indicating that potassium-based emulsifying salts can contribute to sodium reduction while maintaining the technological and sensory characteristics of spreadable processed cheese [43].

Dalmina et al. [102] evaluated reduced-sodium Requeijão cremoso processed cheese manufactured from either fresh sheep milk or sheep milk refrigerated at 4 °C for five days. Replacement of the conventional emulsifying salt with a reduced-sodium formulation decreased the measured sodium content of the final product by 50%. Although mineral composition was modified, no significant changes were detected in color, texture, pH, or the other physicochemical characteristics evaluated. By contrast, refrigeration of raw milk affected curd composition and increased cheese hardness, cohesiveness, and gumminess. The results indicate that emulsifying-salt reformulation can substantially reduce sodium without necessarily compromising the principal quality attributes of Requeijão cremoso, while also demonstrating that raw-material history may independently affect the texture of the final product [102].

From a nutritional perspective, replacing NaCl with other chloride salts changes the mineral profile of cheese rather than simply decreasing its sodium content. Partial substitution with KCl increases potassium intake and may improve the dietary sodium-to-potassium ratio. Potassium-enriched salt substitutes have been associated with reductions in blood pressure and cardiovascular events [103,104]. However, these findings cannot establish universal safety because individuals with serious renal impairment were generally excluded from the relevant trial [104]. Increased potassium intake may elevate the risk of hyperkalemia in susceptible individuals, particularly those with impaired renal potassium excretion or receiving medications that reduce potassium elimination. Substitution with CaCl_2_ or MgCl_2_ similarly increases calcium or magnesium intake. Although calcium and magnesium are physiologically essential, the nutritional consequences of their increased intake depend on the amount added, bioavailability, serving size, background diet, and renal function. Increased calcium intake from CaCl_2_-substituted cheese should not be equated directly with calcium supplementation. Adequate dietary calcium may reduce intestinal oxalate absorption, whereas high-dose calcium supplements, particularly when consumed between meals, may increase urinary calcium excretion and potentially increase nephrolithiasis risk in susceptible individuals [105]. High oral intakes of readily dissociable magnesium salts may cause osmotic diarrhea, while clinically significant magnesium accumulation occurs predominantly when renal clearance is impaired [106]. Therefore, the nutritional implications of mineral-salt substitution should be assessed using the measured elemental sodium, potassium, calcium, and magnesium contents per serving of the final cheese rather than inferred solely from the percentage of NaCl replaced.

Overall, mineral-salt blends broaden the formulation options available for sodium reduction by distributing NaCl replacement among salts with different sensory and technological effects. Combining KCl with CaCl_2_, MgCl_2_, or appropriate flavor-masking components may reduce reliance on high KCl concentrations and thereby limit bitter or metallic notes; however, the resulting effects remain cheese- and concentration-dependent. In particular, divalent ions can modify calcium–casein equilibria, protein hydration, enzyme activity, pH buffering, and texture. In processed cheese, partial replacement of sodium-based emulsifying salts with potassium-based alternatives represents a distinct strategy that can further decrease sodium while maintaining casein dispersion and emulsification when the salt system is appropriately formulated [18,50]. These strategies and their principal technological implications are summarized in Figure 4.

Accordingly, the nominal percentage of NaCl replaced is insufficient to characterize the nutritional outcome of reformulation because it may not correspond directly to the measured reduction in sodium. The final concentrations of sodium, potassium, calcium, and magnesium should therefore be determined analytically and reported separately from NaCl or total salt content. Since mineral substitution may simultaneously affect casein interactions, texture, flavor, microbial stability, and nutritional composition, each formulation requires validation in the specific cheese matrix and assessment against the applicable criteria for sodium-related claims. Where mineral substitution results in unacceptable bitterness or insufficient microbial control, complementary sensory or antimicrobial strategies may be necessary, as discussed in the following section.

### 3.4. Sensory and Microbial Compensation with Functional Ingredients

Reducing NaCl or replacing it with mineral salts may impair flavor perception and weaken microbial inhibition [19]. Functional ingredients have therefore been investigated as complementary interventions to enhance saltiness, mask potassium-associated bitterness, improve flavor complexity, or restore microbial stability. Unlike salt replacers, these ingredients do not necessarily reduce sodium directly; instead, they compensate for specific sensory or microbiological limitations created by cheese reformulation.

Initial research on sensory compensation focused on combining mineral salt replacers with flavor enhancers and bitterness-masking agents. Khetra et al. [50] evaluated the potassium-based commercial salt replacer Saloni K together with hydrolyzed vegetable protein (HVP) as a flavor enhancer and adenosine-5′-monophosphate (AMP) as a bitter blocker in reduced-sodium Cheddar cheese. Preliminary screening identified this combination as producing the greatest saltiness and lowest bitterness. Response-surface optimization subsequently indicated that a formulation with 75% NaCl substitution, 2 g/L HVP, and 300 mg/L AMP provided the most favorable sensory profile. The resulting cheese exhibited sensory properties close to those of the conventional-sodium control, demonstrating that flavor enhancement and bitterness masking can permit relatively extensive NaCl substitution [50]. Building on this combined approach, Silva et al. [107] investigated L-arginine, yeast extract, and oregano extract in probiotic Prato cheese in which 50% of NaCl was replaced with KCl. Quantitative descriptive analysis and temporal dominance of sensations showed that the effects were ingredient specific. Yeast and oregano extracts increased perceived saltiness, while yeast extract also reduced the dominance of KCl-associated bitterness. In contrast, L-arginine produced more extensive changes in the characteristic sensory profile. These findings identify yeast extract as a particularly promising ingredient for simultaneously enhancing saltiness and suppressing bitterness, although the suitability of each flavor enhancer remains dependent on the cheese matrix [107].

Nevertheless, flavor- and culture-based compensation does not always overcome the sensory limitations of sodium reduction. Hoffmann et al. [108] compared direct NaCl reduction with mineral-salt substitution in film-ripened, semi-hard Edam cheese and evaluated adjunct cultures intended to improve flavor formation. The target sodium concentration of ≤0.4 g/100 g, corresponding to approximately ≤1 g NaCl/100 g, was achieved without an evident deterioration in microbiological quality during six weeks of ripening. Salt reduction did not significantly affect the formation of the bitter peptides analyzed. However, the adjunct cultures failed to compensate for the sensory changes, and the reduced-sodium cheeses particularly those containing potassium-based mineral salts remained bitter and organoleptically unsatisfactory. Thus, increased aroma formation or proteolytic activity does not necessarily mask the off-flavors associated with mineral-salt substitution [108].

A similarly mixed outcome was reported by Juan et al. [109], who reduced salt in semi-hard and hard lactose-free cheeses by shortening brining or partially replacing NaCl with KCl at a Na^+^/K^+^ ratio of 60:40. Both approaches achieved reductions exceeding 25% without substantially affecting physicochemical or microbiological properties. However, yeast extract increased free amino acid concentrations while decreasing consumer acceptance because of intensified salty, bitter, and persistent aftertaste sensations. Thus, enhanced formation of flavor-active compounds does not necessarily improve the sensory quality of reduced-sodium cheese [109].

Cross-modal sensory enhancement provides an alternative means of increasing perceived saltiness without adding sodium. Syarifuddin et al. [17] found that salt-associated sardine aroma significantly enhanced saltiness in low-salt cheese, whereas butter aroma produced a weaker effect. Combining both aromas also increased perceived fattiness. These results demonstrate the potential of congruent aroma cues, although their effectiveness depends on aroma–matrix compatibility and consumer associations. Sensory compensation does not address the possible loss of microbial inhibition following NaCl reduction [17]. Taylor and Lathrop [110] found that fermentates and lauric arginate were antimicrobial when applied independently in low-sodium cheese-agar models, but their effectiveness against *Salmonella* spp. and *Escherichia coli* O157:H7 decreased in the presence of certain KCl-containing salt replacers. By contrast, a lactoperoxidase-based system remained effective regardless of the salt substitute. These findings demonstrate that antimicrobial agents and salt replacers must be evaluated in combination, although validation in actual cheese matrices remains necessary [110]. More recently, Lima et al. [84] incorporated 7% (*w*/*w*) babassu (*Attalea speciosa*) mesocarp extract into reduced-sodium Minas fresh cheese containing 0.75% (*w*/*w*) NaCl. The extract reduced bacterial counts and contributed to shelf-life extension, potentially because of its phenolic compounds. However, it also increased hardness and altered cheese color, negatively affecting appearance perception. Babassu extract therefore represents a promising clean-label preservative, but its concentration requires optimization to balance antimicrobial efficacy and sensory quality [84].

Overall, functional ingredients can partly compensate for the sensory and microbial consequences of NaCl reduction. Flavor enhancers and aroma cues may improve perceived saltiness, while plant extracts and enzyme systems may support microbial stability. Nevertheless, their effectiveness is cheese- and formulation-dependent, requiring integrated sensory assessment, shelf-life evaluation, and microbiological challenge testing.

### 3.5. Advanced Processing, Salt-Delivery, and Matrix-Engineering Approaches

Advanced processing strategies complement formulation-based sodium reduction by controlling both the delivery of salt and the response of the cheese matrix to lower NaCl concentrations. Salt-delivery approaches modify particle size, application method, surface distribution, or brining conditions to improve salt-use efficiency, whereas matrix-engineering interventions alter buffering capacity, protein interactions, microbial activity, and ripening behavior. Technologies such as ultrafiltration and high-hydrostatic-pressure processing may consequently help maintain texture, flavor, and microbial stability in reduced-sodium cheese.

Ozturk et al. [111] combined ultrafiltration (UF) retentate fortification, high-hydrostatic-pressure (HHP) processing, and camel chymosin to improve the quality of low-sodium Cheddar cheese. UF retentate was used to increase the buffering capacity of the cheese matrix, HHP to limit excessive starter activity, and camel chymosin to reduce bitterness development. The UF-fortified cheese was treated at 500 MPa for 3 min on the first day after manufacture. HHP reduced starter counts during early ripening and decreased the concentrations of water-soluble nitrogen, trichloroacetic-acid-soluble nitrogen, and β-casein-derived peptides. After one month, all low-sodium cheeses exhibited similar hardness values, which remained lower than that of the conventional-sodium control. Nevertheless, the UF–HHP cheese did not differ significantly from the control in bitterness or acidity. These findings indicate that UF fortification and HHP can regulate acidification and proteolysis, thereby mitigating important quality defects associated with low-sodium Cheddar cheese [111].

Extending the concept of optimized salt delivery, Ferroukhi et al. [42] evaluated alternative dry surface-salting methods in blue-veined cheese by modifying NaCl particle size and application method and by partially substituting NaCl with calcium lactate. Depending on the treatment, sodium content in the cheese core decreased by 11–67%, accompanied by an increase in water activity. Fine-salt sprinkling reduced NaCl content by approximately 45%, whereas partial calcium lactate substitution produced a smaller reduction of approximately 10%; both approaches were considered technically feasible. Microbiological hygiene indicators were not adversely affected, although yeast and mold counts decreased and the richness and composition of the bacterial community changed. Texture and appearance were also modified, demonstrating that changes in salt application and distribution can simultaneously affect sodium diffusion, microbial ecology, and matrix development [42].

In a subsequent investigation of Fourme d’Ambert cheese, Ferroukhi et al. [112] compared NaCl sprinkling with the substitution of 25% of NaCl by calcium lactate during dry salting. Sprinkling reduced sodium content by approximately 35%, whereas calcium lactate substitution achieved a reduction of approximately 47%. The reduced-sodium cheeses exhibited a higher proteolysis index and higher measured concentrations of calcium and vitamins B2, B6, and B12. Although microbiological quality was not markedly compromised, rheological and sensory properties were modified. In particular, the reformulated cheeses were softer and perceived as less salty than the control. These findings confirm that salt-application methods and partial calcium lactate substitution can substantially reduce sodium, but their effects on proteolysis, firmness, and characteristic sensory properties require careful optimization [112].

Overall, advanced processing, salt-delivery optimization, and matrix engineering can either reduce sodium incorporation or compensate for the technological defects associated with low NaCl concentrations. Micronization and sprinkling modify salt dissolution and distribution, whereas UF fortification and HHP regulate buffering capacity, starter activity, and ripening reactions. Nevertheless, these interventions may alter water activity, microbial ecology, proteolysis, texture, and flavor. Their effectiveness must therefore be established separately for each cheese variety, product geometry, salting system, and ripening period. Although these approaches directly modify salt delivery or cheesemaking operations, emerging sodium-reduction strategies increasingly employ real-time sensing, predictive modelling, bioactive preservation, and systems-level analysis to guide cheese-specific reformulation. These next-generation approaches are discussed in Section 4.

## 4. Emerging Approaches to Sodium Reduction

Building on the formulation and processing strategies discussed above, emerging approaches aim to make sodium reduction more targeted, predictive, and cheese-specific (Figure 5). Sensor-based precision salting and AI-assisted process control are being explored to optimize salt uptake and distribution, while clean-label bioactive ingredients may help compensate for the loss of NaCl-mediated microbial inhibition. In parallel, multi-omics analyses can clarify how reformulation affects microbial communities, metabolic pathways, ripening, and flavor development [25,66]. These approaches are particularly relevant because the response of cheese to sodium reduction depends on its composition, manufacturing and salting conditions, starter and adjunct cultures, ripening period, and the spatial distribution of salt within the cheese matrix. Their integration may therefore support the development of reduced-sodium cheeses with improved safety, quality, and sensory consistency, although further validation at pilot and industrial scales remains necessary.

### 4.1. Precision Salting and AI-Assisted Process Control

Conventional salting generally relies on predetermined salt concentrations and brining times. However, variations in cheese size, geometry, moisture, pH, porosity, and protein composition can produce considerable differences in NaCl diffusion and final S/M ratios. Precision salting aims to control this variability by combining real-time measurements of brine conductivity, pH, temperature, total dissolved solids, and cheese composition with models describing salt and moisture transfer [66]. Such systems could support the adjustment of brining time and composition according to the characteristics of each cheese batch, thereby preventing both excessive sodium incorporation and insufficient salting.

Artificial intelligence and machine-learning models provide additional opportunities for the optimization of these processes [25]. Artificial neural networks have already been used to predict the physicochemical properties of brines and the physical, textural, and sensory properties of white brined cheese produced with partial replacement of NaCl by calcium citrate or calcium lactate [24]. These models successfully associated salt composition, brine properties, and storage time with cheese hardness, chewiness, breakage, and overall sensory evaluation. AI-assisted models may therefore reduce the number of experimental trials required during product development and help identify combinations of NaCl concentration, substitute salts, brining conditions, and storage parameters that produce acceptable low-sodium cheese. Nevertheless, their industrial application requires validation using larger datasets obtained from different cheese varieties, manufacturing scales, and ripening conditions.

### 4.2. Clean-Label Bioactive Ingredients

The reduction in NaCl may weaken the natural microbial stability of cheese, increasing the need for complementary preservation strategies. In this context, plant extracts, essential oils, and food-processing by-products containing phenolic and antimicrobial compounds are receiving increasing attention as clean-label ingredients [21,22,30]. These materials may partially compensate for the reduction in the bacteriostatic activity of NaCl while also providing antioxidant activity and potentially improving the functional value of the final product.

The incorporation of babassu mesocarp extract into reduced-sodium Minas fresh cheese has been reported to decrease viable counts of the bacterial populations evaluated, an antimicrobial effect attributed primarily to the extract’s phenolic constituents [84]. More recently, phenolic-rich ethanolic extracts obtained from globe artichoke leaves were evaluated in low-salt Domiati cheese. The extract exhibited broad antimicrobial activity against several foodborne pathogens and improved the physicochemical, microbiological, antioxidant, and storage characteristics of the cheese during refrigerated storage [22]. This approach is particularly interesting from a sustainability perspective because artichoke leaves represent an agricultural by-product that can be valorized as a functional food ingredient.

Essential oils may also be used directly in cheese or brine as natural post-processing preservatives. Caraway essential oil, which is rich in carvone and limonene, demonstrated antimicrobial activity against Escherichia coli and Staphylococcus aureus in low-salt white brined cheese. A concentration of 0.12% (v/v) completely inhibited *E. coli*, whereas concentrations of 0.25% (v/v) produced a bactericidal effect against *S. aureus* without adversely affecting the reported sensory characteristics of the cheese [21]. Despite these promising findings, the industrial use of plant extracts and essential oils requires careful standardization because their composition may vary according to plant variety, geographical origin, extraction procedure, and storage conditions. Their effective antimicrobial concentrations must also be balanced against possible changes in cheese flavor, aroma, color, starter-culture activity, and consumer acceptance.

### 4.3. Multi-Omics-Guided Reformulation

Multi-omics technologies are not sodium-reduction methods themselves, but they provide a powerful framework for designing and validating reduced-sodium cheeses [66]. Metagenomics and metatranscriptomics can reveal changes in microbial community composition and activity, while metabolomics, volatilomics, and proteomics can identify alterations in flavor compounds, amino acids, organic acids, lipids, peptides, and potential spoilage or safety markers. Integration of these datasets with physicochemical and sensory measurements can clarify why a particular sodium-reduction strategy succeeds in one cheese variety but produces undesirable effects in another.

Previous metagenomic and metatranscriptomic analyses have demonstrated that the microbiological consequences of NaCl reduction or substitution are strongly dependent on cheese type [57]. More recently, an integrated analysis of Cheddar cheeses containing 2.0%, 1.5%, or 1.0% salt combined physicochemical measurements, volatile compound analysis, 16S rRNA gene sequencing, and untargeted metabolomics. Cheddar cheese containing 1.5% salt retained microbial and metabolic characteristics relatively similar to those of the full-salt product. In contrast, reduction to 1.0% produced more pronounced changes in texture and microbial community structure, including increased abundance of genera associated with spoilage risk, together with substantial changes in amino-acid metabolism and volatile-compound production [113]. These results demonstrate that multi-omics analysis can help identify the point at which sodium reduction begins to compromise microbial stability or alter the characteristic flavor profile of a cheese.

The combination of multi-omics data with AI-assisted predictive models may eventually enable cheese-specific sodium-reduction strategies. Such models could integrate salt concentration, moisture, pH, water activity, microbial composition, metabolite profiles, processing parameters, and sensory responses to predict the lowest technologically safe and sensorially acceptable sodium level for each cheese variety. Future studies should focus on industrial-scale validation, standardized analytical protocols, regulatory acceptance of new ingredients, and the development of sufficiently large and diverse datasets for reliable predictive modeling.

### 4.4. Industrial Implementation and Regulatory Considerations

The translation of sodium-reduction strategies from experimental studies to commercial cheese production remains insufficiently documented. Most investigations have been conducted at laboratory or pilot scale and have primarily evaluated physicochemical, microbiological, textural, and sensory properties [24,101]. Evidence therefore remains limited concerning batch-to-batch reproducibility, brine recycling, the accumulation of potassium, calcium, or magnesium during repeated processing cycles, equipment corrosion and material compatibility, wastewater composition, ingredient and processing costs, life-cycle impacts, and consumer acceptance following repeated consumption rather than a single sensory session. Consequently, technological feasibility demonstrated in individual production trials cannot be considered sufficient evidence of economic viability, environmental sustainability, or long-term consumer acceptance at industrial scale. Industrial validation should include repeated production batches and measurements of product yield, processing time, energy and water consumption, shelf-life, process variability, and total reformulation cost, including any additional cultures, antimicrobial systems, or flavor-masking ingredients required.

Brine management represents a particular implementation challenge. Repeated use of brines containing NaCl substitutes may progressively modify the concentrations of Na^+^, K^+^, Ca^2+^, Mg^2+^, and Cl^−^ through mineral exchange with the cheese. Simultaneously, the transfer of moisture, lactose, lactate, soluble proteins, and microorganisms from the cheese into the brine may alter its physicochemical and microbiological properties. These changes could affect mineral equilibria, salt uptake, cheese texture, microbial stability, and process reproducibility. Recycled brines should therefore be monitored through ion-specific mineral analysis together with measurements of total salt concentration, pH, organic load, and microbial quality. Eykens et al. (2018) demonstrated the technical feasibility of concentrating and recovering cheese brines by membrane distillation at laboratory and pilot scales [114]. Similarly, Kezia et al. (2015) evaluated direct-contact membrane distillation for concentrating saline dairy effluent [115]. Although these studies demonstrate the technical potential of membrane-based brine and wastewater management, they do not establish the economic performance, energy efficiency, or long-term operational stability of these systems during repeated industrial use.

Replacement of NaCl with KCl, CaCl_2_, or MgCl_2_ changes the ionic composition of brines and wastewater without necessarily decreasing their chloride load. Sodium reduction should therefore not be assumed to reduce chloride-related deterioration of processing equipment. Compatibility with stainless-steel grades, welded surfaces, seals, pumps, and cleaning systems should be evaluated under the chloride concentration, pH, temperature, and cleaning conditions encountered in the production facility. Wastewater characterization should similarly include Na^+^, K^+^, Ca^2+^, Mg^2+^, Cl^−^, salinity, and chemical oxygen demand before environmental benefits are claimed. These factors should currently be regarded as industrial validation requirements because reduced-sodium cheese studies have rarely quantified corrosion, equipment lifetime, wastewater treatability, or life-cycle environmental effects.

Clean-label expectations introduce an additional constraint because consumer responses are influenced by ingredient familiarity, label terminology, processing transparency, and the sensory characteristics of the final product. Fernandes et al. (2025) emphasized that clean-label cheese development must balance consumer expectations with microbiological safety, technological functionality, and product quality [116]. Natural extracts and other clean-label ingredients may also exhibit compositional variability related to their biological source, extraction method, and storage conditions, requiring chemical and functional standardization before industrial application.

Regulatory eligibility must be determined from the analytically measured sodium content of the final cheese rather than from the nominal percentage of NaCl replaced. Kurćubić et al. (2024), for example, reported a sodium concentration of 334.80 ± 24.60 mg/100 g after complete replacement of NaCl with a Na/K mineral blend [101]. Although this represented a substantial comparative sodium reduction, the cheese did not meet the applicable criterion for classification as a “low-sodium” product. Within the European Union, comparative sodium- or salt-reduction claims must comply with Regulation (EC) No 1924/2006 [8], while the technological use and specifications of mineral salts must be evaluated according to Regulation (EC) No 1333/2008 [92] and Commission Regulation (EU) No 231/2012 [91]. Compliance should also be assessed against cheese-specific compositional standards and the product specifications of cheeses registered under protected designation of origin or protected geographical indication schemes. Industrial implementation therefore requires integrated technological, economic, environmental, sensory, and regulatory validation rather than extrapolation from the percentage of NaCl replacement alone.

## 5. Conclusions

Current evidence supports moderate, cheese-specific NaCl reduction and partial replacement with KCl as the most technically established approaches. However, the available studies differ substantially in cheese composition, salting method, ripening time, analytical measurements, and definition of “reduced sodium.” Many studies report the percentage of NaCl removed from the formulation without measuring the resulting sodium concentration, salt-in-moisture ratio, water activity, or spatial salt distribution. This prevents direct comparison among products and limits identification of the minimum NaCl concentration required for technological and microbiological stability.

Several important evidence gaps remain. Pathogen-challenge studies covering the complete commercial shelf life are scarce, particularly for fresh, surface-ripened, raw-milk, and very-low-sodium cheeses. The effects of NaCl reduction on lipolysis, amino-acid catabolism, volatile-compound formation, and microbial interactions are less thoroughly characterized than its effects on moisture, proteolysis, and texture. The most promising research direction is therefore the development of cheese-specific formulation limits supported by integrated measurements of sodium, total chloride salts, moisture, salt-in-moisture ratio, water activity, pH, organic acids, spatial salt gradients, microbial succession, metabolites, texture, and sensory acceptance. Studies should combine pathogen-challenge testing with metagenomics, metabolomics, volatilomics, and conventional culture-based measurements to identify causal links between reformulation, microbial ecology, and product safety. Precision salting, real-time brine monitoring, selected starter and protective cultures, and predictive models are promising tools, but they should be validated using independent pilot- and industrial-scale batches. Future work should ultimately define the lowest sodium concentration that remains microbiologically safe, technologically reproducible, sensorially acceptable, legally compliant, and economically feasible for each major cheese category.

## Figures and Tables

**Figure 1:**
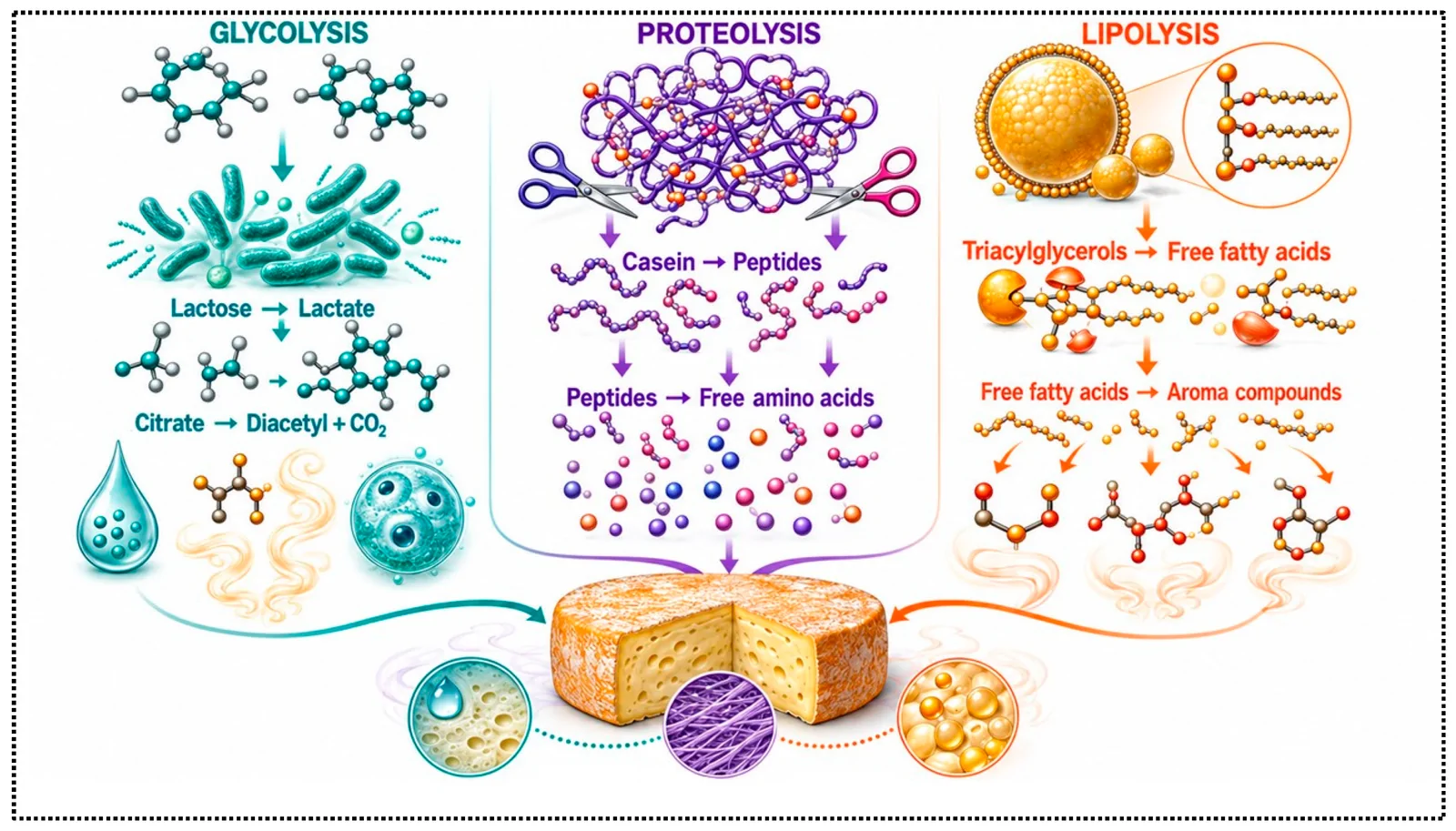
Principal biochemical pathways involved in cheese ripening and their contributions to ripened-cheese quality. Abbreviation: CO_2_, carbon dioxide.

**Figure 2:**
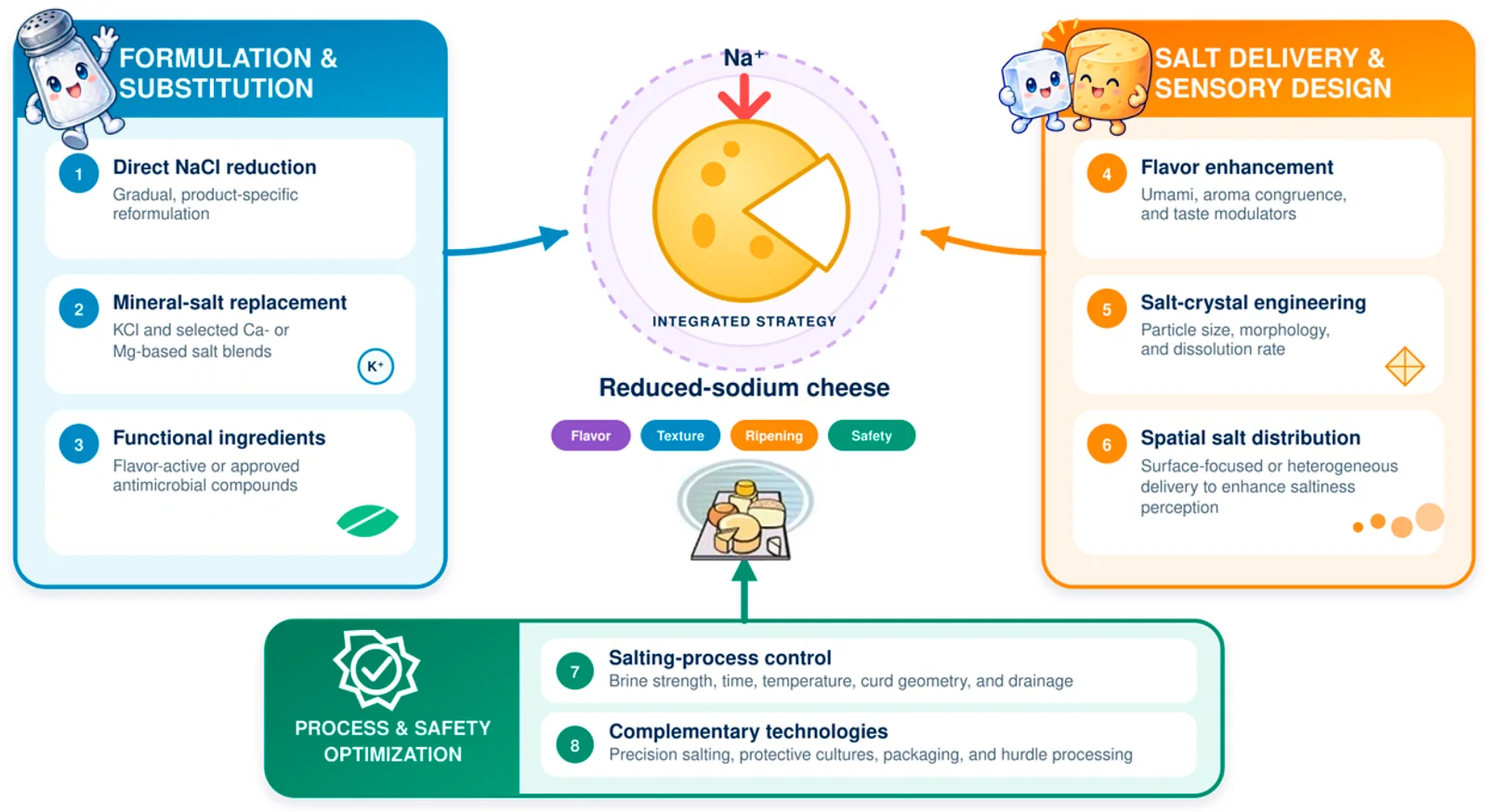
Integrated formulation, sensory-design, and process-control strategies for sodium reduction in cheese and their combined contribution to flavor, texture, ripening, safety, and consumer acceptability. Abbreviations: NaCl, sodium chloride; KCl, potassium chloride; Na^+^, sodium ion; K^+^, potassium ion; Ca, calcium; Mg, magnesium.

**Figure 3:**
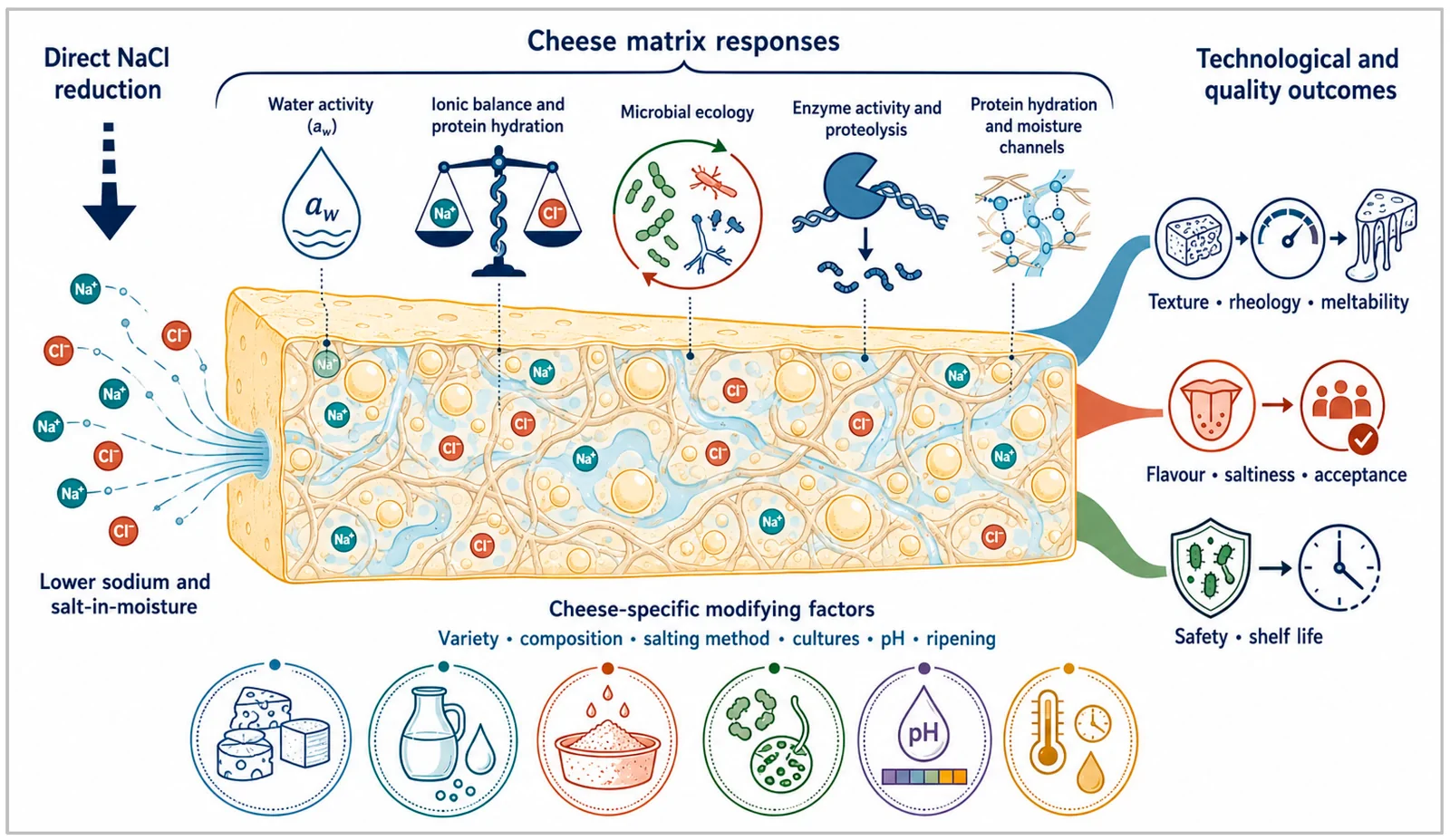
Interconnected physicochemical, microbiological, and technological consequences of direct NaCl reduction in cheese.

**Figure 4:**
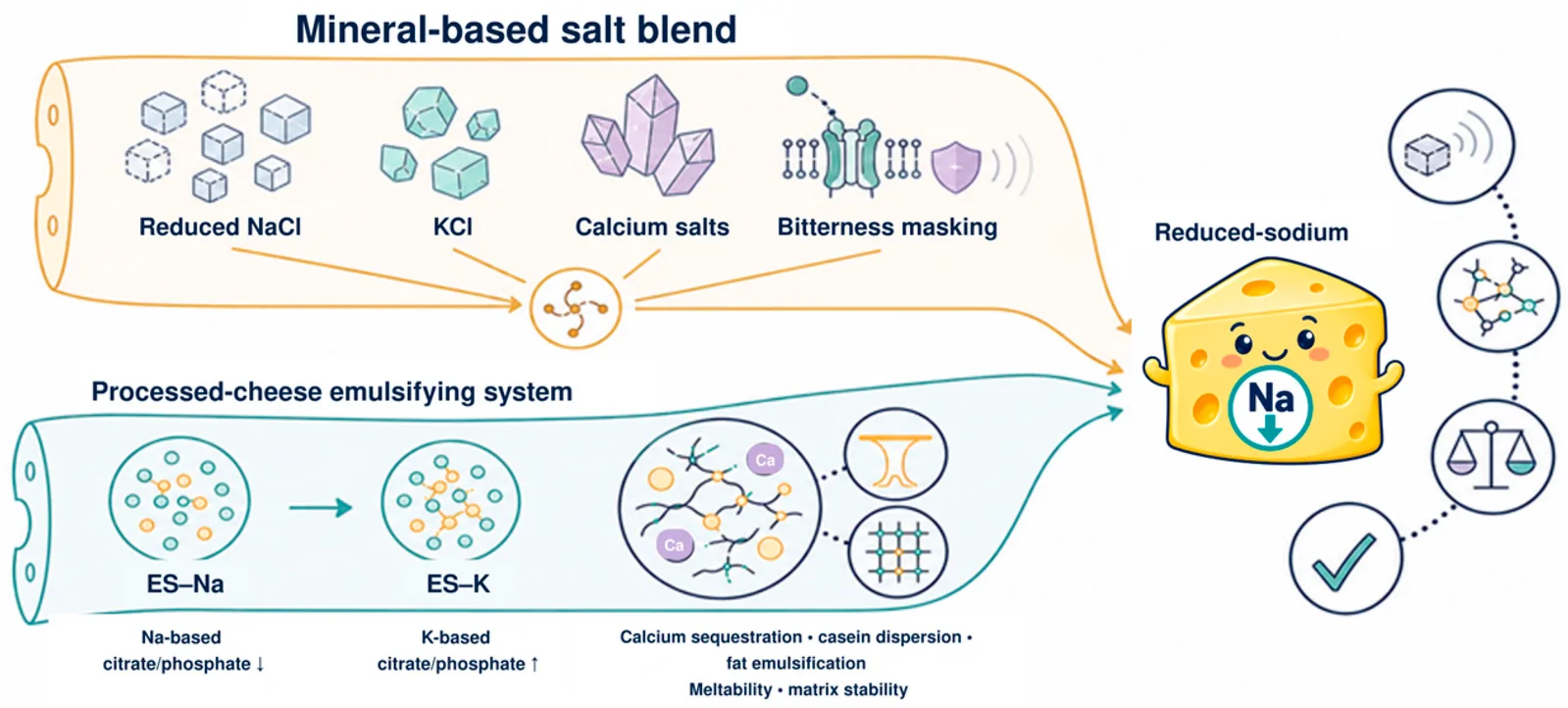
Mineral-based salt blends and low-sodium emulsifying systems for sodium reduction in cheese.

**Figure 5:**
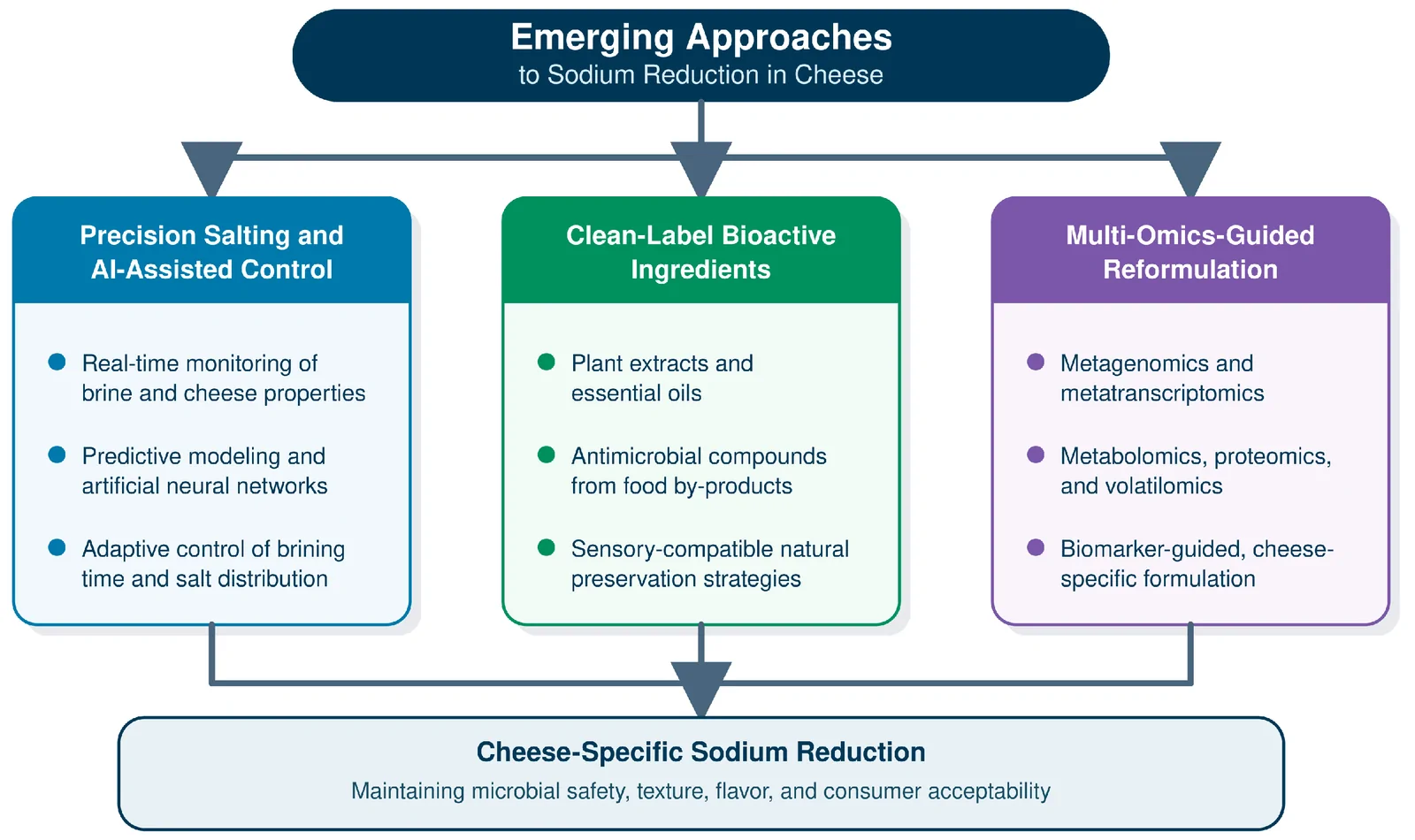
Advanced strategies for developing reduced-sodium cheese.

**Table 1:** Representative sodium content, salt equivalents, and technological functions of salt in different cheese types.

Cheese Type	Literature Sodium Interval (mg/100 g)	Equivalent Salt * (g NaCl/100 g)	USDA Reference Sodium Value (mg/100 g)	Technological Role of Salt	References
Cheddar (natural)	474–731	1.19–1.83	653	Controls moisture and water activity and regulates microbial activity, proteolysis, texture, flavor development, and microbial stability	[5,31,32]
Gouda	368–968	0.92–2.42	819	Flavor, texture, enzyme activity, and microbial control	[31,33,34]
Brie-type cheese	91–731	0.23–1.83	629	Flavor, ripening, regulates surface microbial succession, texture, aroma formation, microbial control, and biogenic-amine production	[31,35,36]
Feta PDO	480–2040	1.20–5.10	1140	Preservation during brining, flavor, texture, ripening and microbial stability	[16,28,31,37,38]
Kalathaki Limnou PDO	1188–1452	2.97–3.63	Not available	Brine preservation, regulation of water activity, proteolysis microbial growth, and ripening	[28,37]
Low-moisture part-skim Mozzarella	452–876	1.13–2.19	666	Controls moisture, protein hydration, proteolysis, rheology, meltability, flowability, and stretchability	[31,32,39]
Cottage Cheese	164–276	0.41–0.69	315	Texture, rheological behavior, and sensory properties	[31,40,41]
Blue Cheese	672–1200	1.68–3.00	1150	Controls salt diffusion, water activity, microbial-community structure, mold and yeast development, texture, appearance, and flavor formation	[31,41,42]
Process cheese singles	936–1590	2.34–3.98	1670	NaCl contributes saltiness and preservation, whereas sodium-based emulsifying salts control casein dispersion, emulsification, pH, texture, melting, and stability	[31,32,43,44]
Katiki Domokou	400	1.00	Not available	Flavor, moisture regulation, and microbial control	[45]
Kefalograviera PDO	1220–1588	3.05–3.97	Not available	Brining and dry salting regulate water activity, ripening, microbial stability, texture, and sensory quality	[37,46]

* Salt equivalents were calculated from sodium content according to the conventional nutrition-labelling relationship: salt (g/100 g) = sodium (mg/100 g) × 2.5/1000. Where only salt or NaCl content was reported, the corresponding sodium equivalent was calculated as sodium (mg/100 g) = salt (g/100 g) × 1000/2.5. Converted values represent salt or sodium equivalents and not independent analytical measurements. The literature values are representative of the products and experimental conditions examined and may vary according to cheese composition, salting method, moisture content, manufacturing conditions, and ripening stage. The technological functions listed are the principal roles of salt and are not exhaustive. USDA reference values were obtained from FoodData Central SR Legacy [31] and represent generic U.S. food-composition entries rather than universal averages or market-wide sodium ranges. Depending on the entry, these values may represent analytical composites, label-derived estimates, or imputed data. In particular, the generic USDA Feta entry should not be interpreted as a compositional specification for Feta PDO or other protected cheeses. Literature-derived intervals and USDA reference values are therefore presented separately.

## Data Availability

No new data were created or analyzed in this study. Data sharing is not applicable to this article.
